# Pediatric endocrine disorders: a review of intracranial findings and appropriate imaging

**DOI:** 10.1007/s00247-026-06626-x

**Published:** 2026-05-11

**Authors:** Alankrit Shatadal, Kimberly K. Vidmar, David B. Allen, Teresa Chapman

**Affiliations:** 1 School of Medicine and Public Health, University of Wisconsin–Madison, Madison, USA; 2 Division of Pediatric Endocrinology and Diabetes, Department of Pediatrics, University of Wisconsin–Madison, Madison, USA; 3 Department of Radiology, University of Wisconsin–Madison, Madison, USA; 4 Professor, Pediatric Radiology University of Wisconsin School of Medicine & Public Health, 600 Highland Ave, Madison, WI 53792 USA

**Keywords:** Brain MRI, Diabetes insipidus, Diabetes mellitus, Intrasellar, Hypothalamus, Pituitary

## Abstract

Endocrine disorders in pediatric patients often involve a variety of imaging modalities as part of diagnostic workups or disease surveillance. This pictorial essay and review of the literature highlights a wide range of imaging findings for central nervous system pathologies leading to endocrine-related diseases, as well as for glucose metabolism disorders that impact the brain. In this review focusing on the central nervous system, congenital, developmental, inflammatory, and neoplastic disorders of the pituitary and hypothalamus will be discussed, as well as brain findings that may be seen with diabetic ketoacidosis and with congenital hypoglycemia. Differential diagnostic considerations, appropriate imaging protocols, and clinical management strategies will be described.

**Graphical Abstract:**

**Figure:**
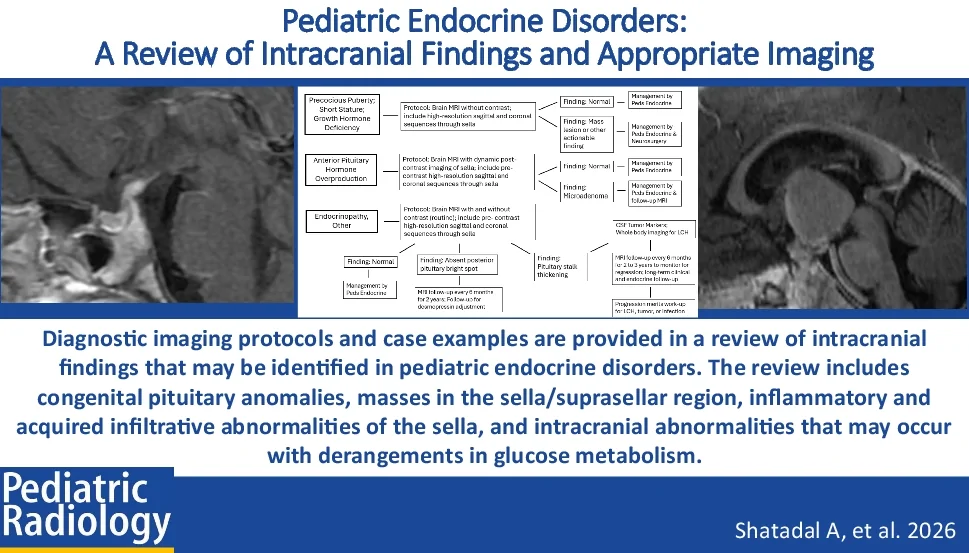

## Introduction

Pediatric endocrinologists specialize in disorders of the endocrine system in infants, children and adolescents, managing conditions related to hormones, growth, puberty, and metabolism. A frequent focus in this field is the hypothalamic-pituitary axis, a crucial central neuroendocrine system that regulates hormone control. Midline congenital brain anomalies such as agenesis of the corpus callosum and optic nerve hypoplasia are indications that the hypothalamic-pituitary axis development may not be normal. Endocrinopathies that may arise in the setting of midline brain anomalies include diabetes insipidus, growth hormone deficiency, central adrenal insufficiency, hypogonadotropic hypogonadism, and multiple pituitary deficiencies (or panhypopituitarism) [1]. Apart from major midline brain malformations, isolated maldevelopment of the adenohypophysis or neurohypophysis may manifest with growth hormone deficiency or multiple pituitary hormone deficiencies.

In addition to congenital intracranial midline anomalies, acquired intracranial diseases may lead to hormone deficiencies or excesses requiring the attention of a pediatric endocrinologist and consultation by a pediatric neuroradiologist. For example, intracranial masses involving the hypothalamus or pituitary will require multidisciplinary care including pediatric endocrinology. Type 1 and type 2 diabetes mellitus are additional conditions that sometimes intersect with diagnostic neuroimaging when complications arise.

Neuroimaging for pediatric endocrinopathies most commonly relies on brain magnetic resonance imaging (MRI) to assess for suspected intracranial abnormalities, although in some situations, computed tomography (CT) of the head is appropriate. MRI of the sellar/suprasellar region can be performed at either 1.5-T or 3-T magnet strength. Non-contrast T1-weighted and T2-weighted sequences in both the sagittal and the coronal planes are important for evaluating the sella and suprasellar space. In addition, an isotropic high-resolution, sagittal heavily T2-weighted sequence provides excellent anatomic detail of the pituitary stalk and hypothalamic region. Evaluation of the full brain should always be performed in the setting of endocrine abnormalities to search for additional findings beyond the sella/suprasellar region. Post-contrast imaging using gadolinium may be indicated [2].

In this review, appropriate imaging for both common and rare disorders evaluated by the pediatric endocrinologist will be discussed, along with case examples demonstrating pertinent imaging findings. Congenital anomalies covered include abnormal development of the hypothalamic-pituitary axis, isolated pituitary findings, and hypogonadotropic hypogonadism (Kallman syndrome). Acquired disorders include benign and malignant neoplasms involving the sella and hypothalamus, pituitary adenomas, and inflammatory or infectious diseases involving the pituitary and hypothalamus. Finally, intracranial abnormalities that arise from disorders of glucose metabolism will be reviewed.

## Pituitary development and anatomy on brain MRI

**Fig. 1:**
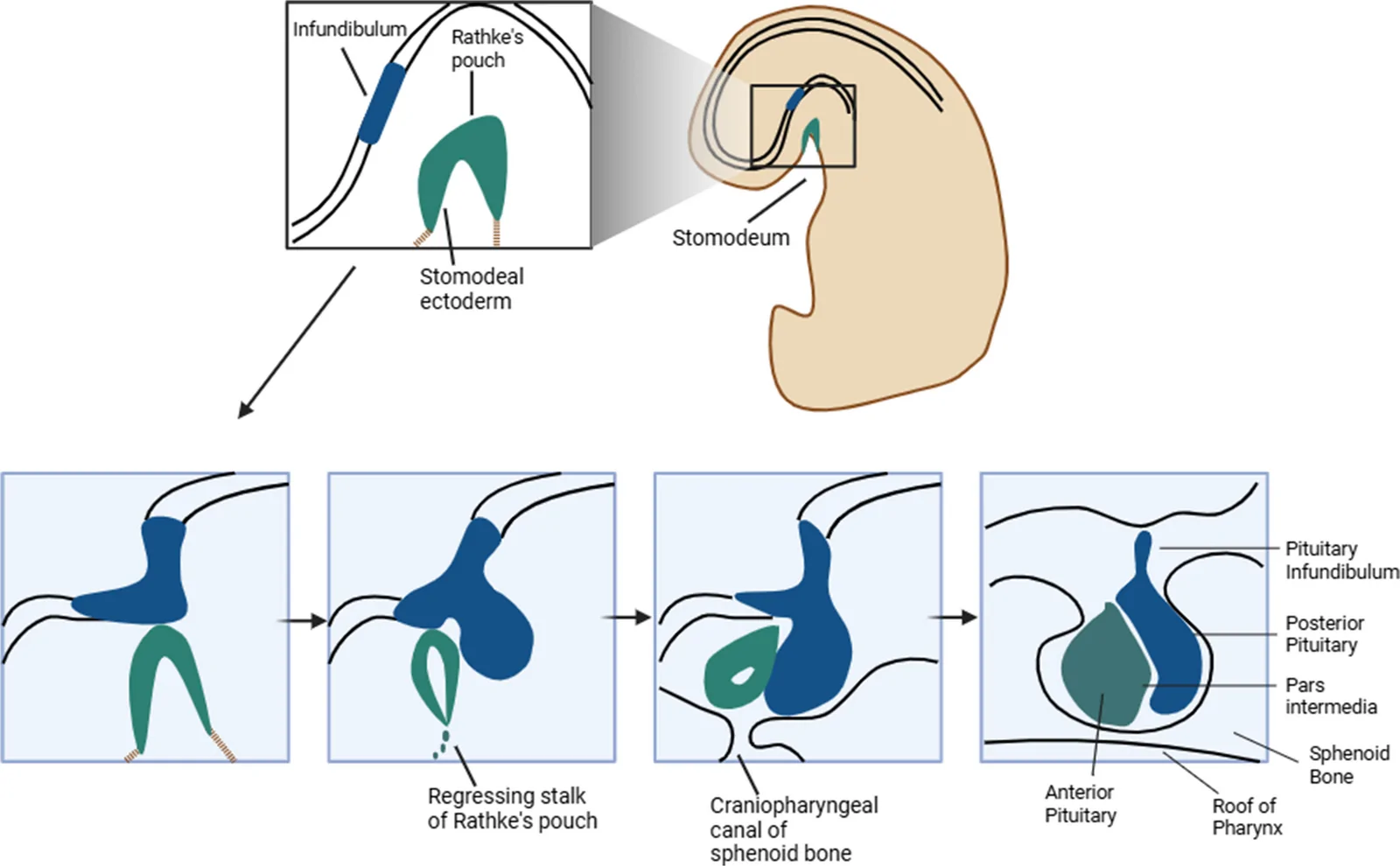
Embryologic development of the pituitary. During the third week of embryological development, a diverticulum (the infundibulum) forms in the floor of the third ventricle and grows ventrally toward the stomodeum. At the same time, a plate of ectoderm appears in the roof of the stomodeum and invaginates to form Rathke’s pouch, which grows dorsally toward the infundibulum. The connecting stalk between the stomodeum and Rathke’s pouch eventually regresses and forms a sac that is apposed to the cranial surface of the infundibulum. This then differentiates to form the adenohypophysis of the pituitary, which gives rise to the anterior lobe of the pituitary and to the pars intermedia. The distal portion of the infundibulum meanwhile differentiates to form the neurohypophysis (posterior pituitary). Adapted from Fig. 13−13 in Larsen WJ (1993) [4]

**Fig. 2:**
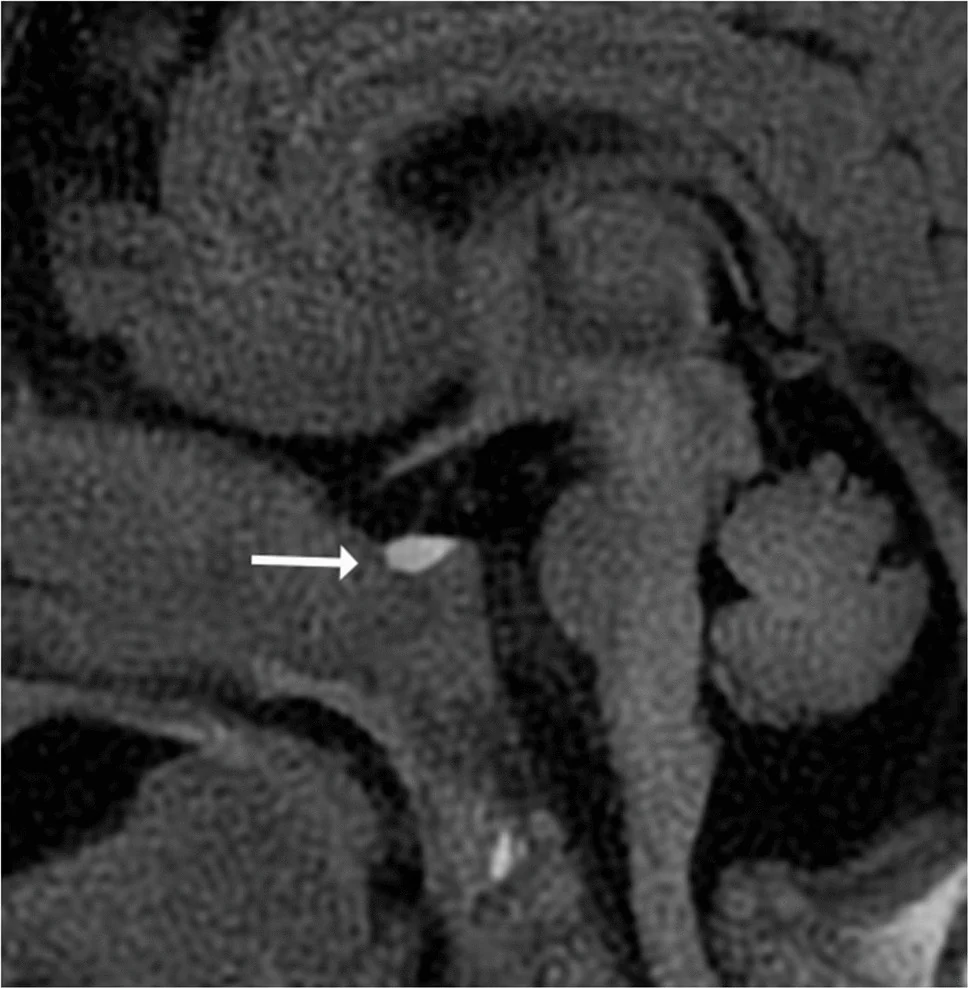
Normal appearance of adenohypophysis in a 29-day-old male infant with seizures (no endocrinopathy). Sagittal T1-weighted brain magnetic resonance imaging focused on the pituitary gland demonstrates a uniformly hyperintense adenohypophysis (*arrow*)

**Fig. 3:**
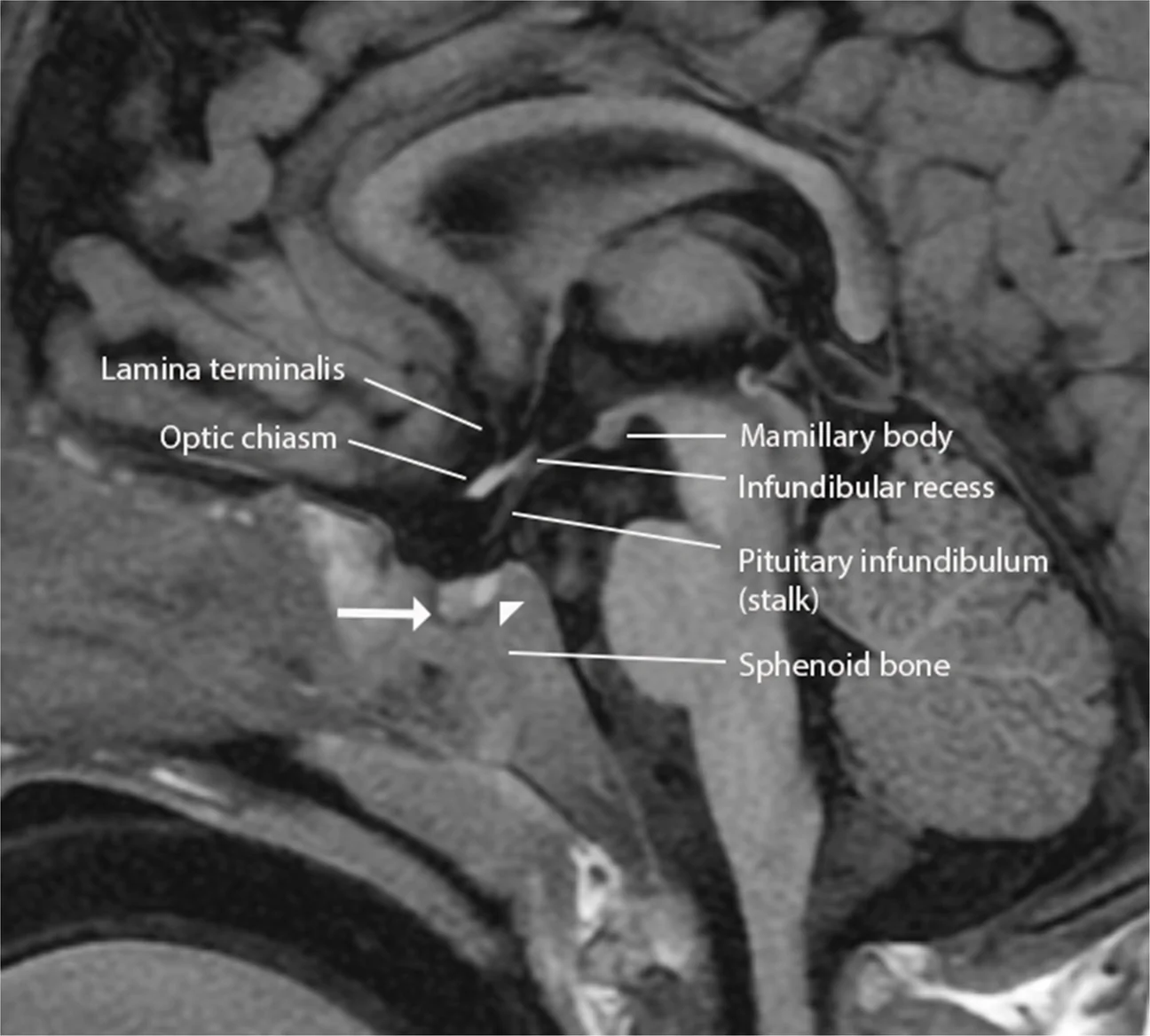
Normal appearance of adenohypophysis in a 6-month-old male infant with seizure (no endocrinopathy). Sagittal T1-weighted brain magnetic resonance imaging focused on the pituitary gland demonstrates an isointense adenohypophysis (*arrow*) distinguishable from the hyperintense posterior pituitary bright spot (*arrowhead*). Additional anatomic structures of the hypothalamic region are labeled

Before discussing pituitary anomalies, a fundamental understanding of typical pituitary embryology, function, and appearance on diagnostic imaging is imperative. The pituitary gland forms early in embryonic development within the sella turcica, a depression within the central body of the sphenoid bone. The anterior pituitary gland (adenohypophysis) arises from an invagination of the oral ectoderm and forms Rathke’s pouch, whereas the posterior pituitary gland (neurohypophysis) originates from the neuroectoderm (Fig. 1) [3, 4]. The normal anterior pituitary gland (a true secretory gland responsible for secreting growth hormone, gonadotropins, thyroid-stimulating hormone, prolactin, and adrenocorticotropin) is uniformly isointense on T1-weighted imaging and measures 3–10 mm in craniocaudal height [5]. Pituitary gland size tends to be slightly higher in females than in males [5, 6]. Furthermore, it is recognized that in adolescence there is physiologic enlargement of the anterior pituitary lobe manifesting with a rounded, convex superior border when viewed in the sagittal plane on brain MRI [7]. Of note, in the newborn infant, the anterior pituitary is transiently hyperintense on T1-weighted imaging (Fig. 2), perhaps due to circulating placental estrogen effects causing hyperplasia of prolactin-producing cells (lactotrophs) [8]. By approximately 3 months to 4 months of age in infancy, this effect resolves, and the gland appears isointense through adolescence (Fig. 3).

The posterior pituitary gland is formed by the evagination of neural tissue from the floor of the third ventricle and contains axons extending inferiorly from the hypothalamus (Fig. 1). Both vasopressin and oxytocin are synthesized by neurons in the supraoptic and paraventricular nuclei of the hypothalamus and transported in storage vesicles to the posterior pituitary, from where they are ultimately released into the systemic circulation through fenestrated capillaries around the pituitary gland [9, 10]. The appearance of the posterior pituitary gland (responsible for secretion of vasopressin, also known as antidiuretic hormone, and oxytocin) is hyperintense on T1-weighted MR imaging in infants, children, and adolescents and is referred to as the posterior pituitary “bright spot” (Fig. 3). This is postulated to be due to the paramagnetic effects of vasopressin or its carrier proteins [11].

### Hypothalamic-pituitary developmental disorders

**Fig. 4:**
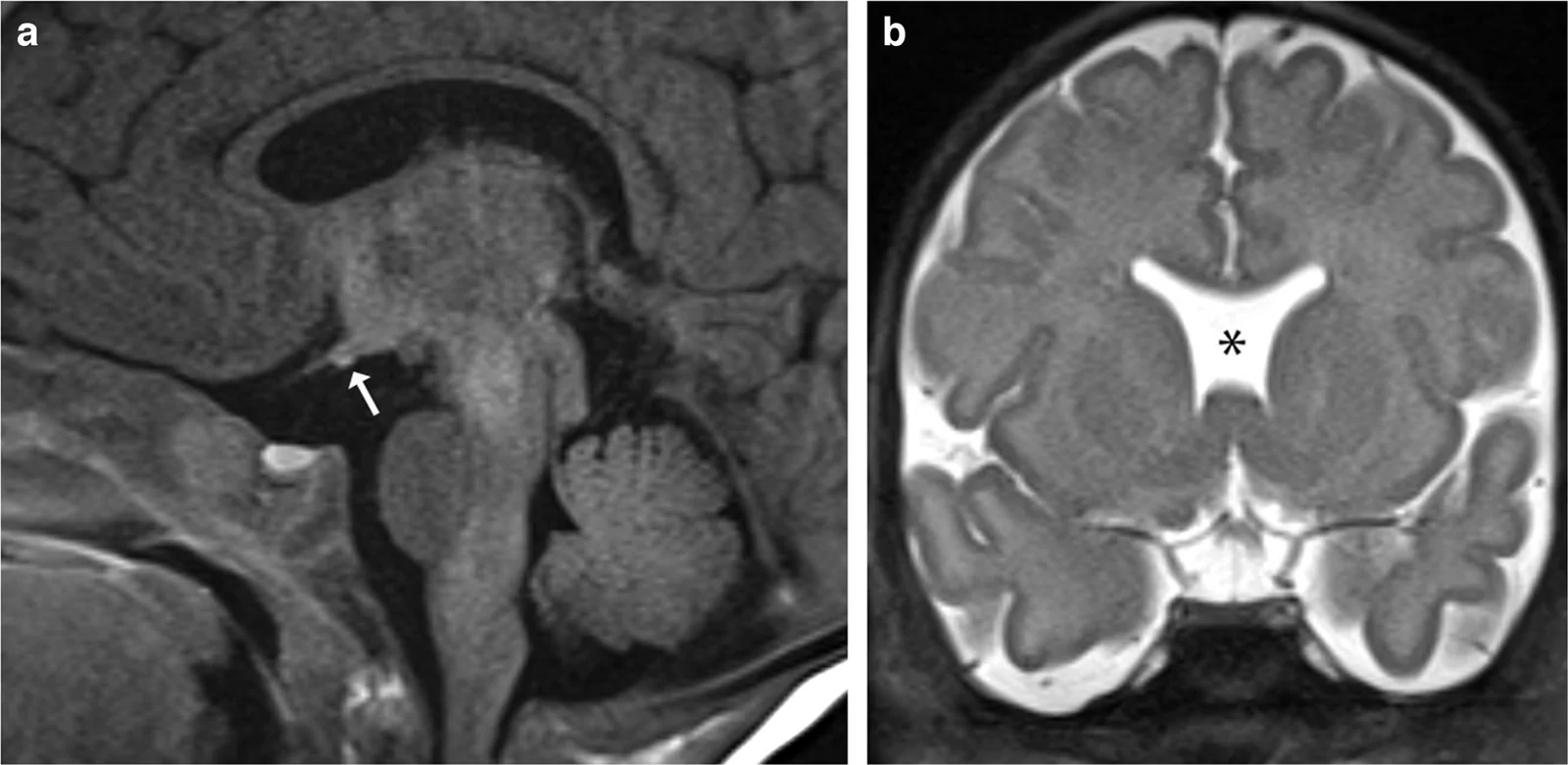
Twelve-day-old male infant with a prenatal diagnosis of absence of the septum pellucidum and postnatal diagnoses of bilateral optic nerve hypoplasia, central hypothyroidism and partial adrenal insufficiency. **a** Sagittal T1-weighted midline image focused on the sella shows an ectopic neurohypophysis positioned at the median eminence of the hypothalamus (*arrow*). **b** Coronal T2-weighted brain magnetic resonance image shows absence of the septum pellucidum (*asterisk*)

**Fig. 5:**
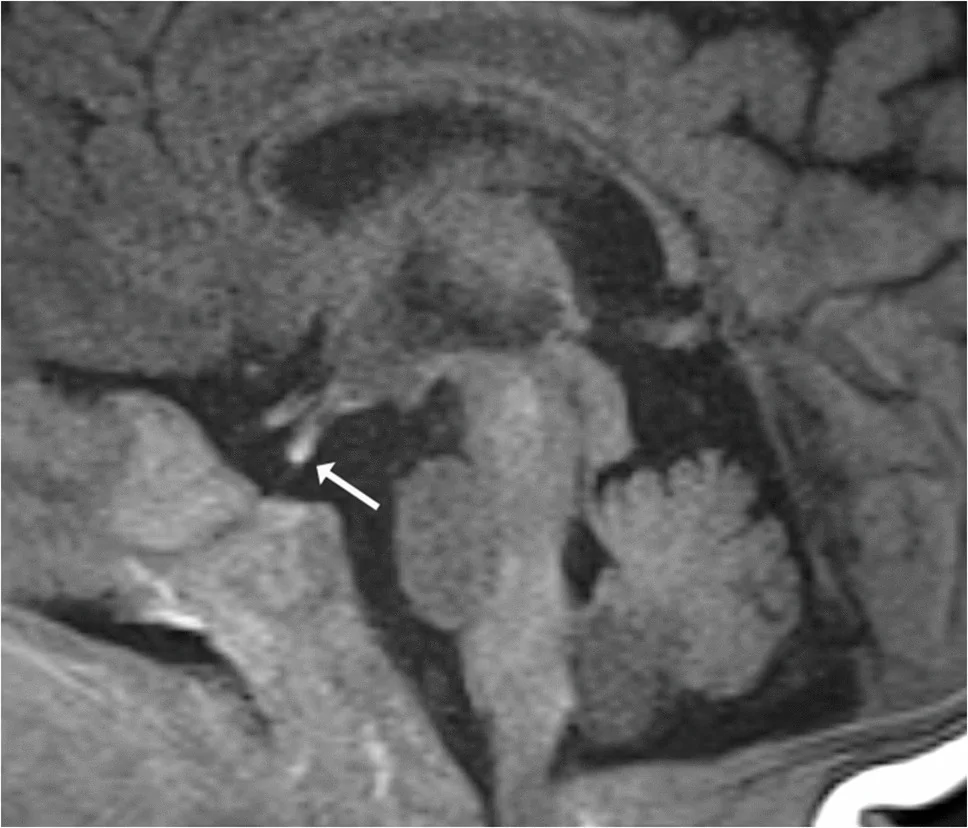
Two-month-old male infant with a history of prematurity and congenital heart disease, incidentally discovered to have ectopic posterior pituitary on brain imaging performed for neurodevelopmental prognostic reasons. Sagittal T1-weighted image focused on the region of the hypothalamus unexpectedly identifies an ectopic neurohypophysis positioned midway along the pituitary stalk between the hypothalamus and sella (*arrow*). A full panel of endocrine laboratory studies was normal at both 2 months and 4 months of age

**Fig. 6:**
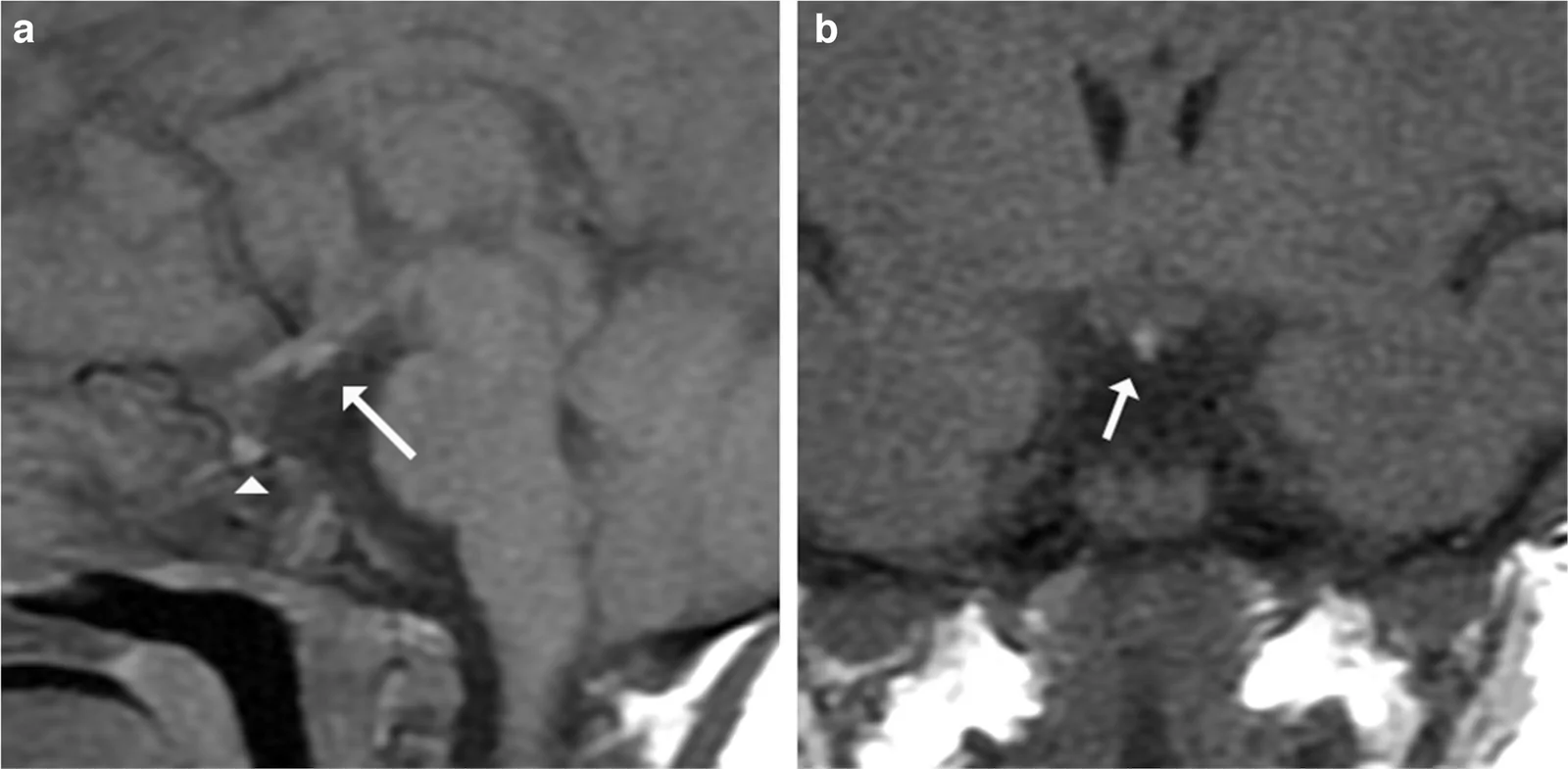
One-month-old female infant with multiple pituitary hormone deficiencies and pituitary stalk interruption syndrome. **a** Sagittal and (**b**) coronal T1-weighted images through the hypothalamus and sella show an ectopic posterior pituitary positioned at the median eminence of the hypothalamus (*arrow*), absence of the pituitary stalk, and hypoplasia of the adenohypophysis (*arrowhead*)

Abnormal posterior pituitary migration may occur, resulting in ectopia of the posterior pituitary bright spot appearing at the median eminence of the hypothalamus (Fig. 4) or along the pituitary stalk (Fig. 5). Ectopic posterior pituitary has been associated with various gene mutations including GLI2, PROP1, LHX4, IFT172, SOX3, OTX2, and HESX1, as well as with midline brain malformations [12–16]. Even rarer than ectopia of the posterior pituitary is partial ectopia, characterized by two distinct bright spots, one in orthotopic position within the sella and one in an abnormal position [13]. Overlapping with the diagnosis of ectopic posterior pituitary is the pituitary stalk interruption syndrome, which refers to a thin, interrupted or absent pituitary stalk, hypoplasia of the adenohypophysis, and an ectopic posterior pituitary (Fig. 6) [17–19]. This is an extremely rare malformation, estimated to affect five in every million live births [20]. The proposed etiology of this condition points to mutations in the Wnt, Notch, and SHH signaling pathways [17]. Structural differences of the hypothalamic-pituitary region on MRI such as ectopic posterior pituitary and pituitary stalk interruption have been associated with isolated growth hormone deficiency and less commonly combined pituitary hormone deficiencies with variable timing of onset and progression of deficiencies and symptoms. Patients with these differences in anatomy noted incidentally (Fig. 5) should have all pituitary hormones screened at diagnosis and at a regular interval thereafter, or sooner if signs or symptoms develop. Patients may present with hypoglycemia, micropenis, cryptorchidism, or jaundice in the neonatal period or later with short stature or delayed puberty, depending on the specific hormone deficiencies present. The mainstay of treatment is prompt and appropriate hormone replacement [20].

A subtle, uncommon congenital midline anomaly relevant to this anatomy with unclear impact upon neuroendocrine function is the interhypothalamic adhesion, which refers to horizontally oriented parenchymal bands of tissue that structurally connect the periventricular hypothalamic zones and the hypothalamic nuclei [21]. These adhesions may signify a focal area of failed hypothalamic separation. They can be visualized in cross-section on the sagittal plane as a characteristic nodule of tissue in the mid-anterior third ventricle [21]. When observed, correlation with neuroendocrine function is warranted.

**Fig. 7:**
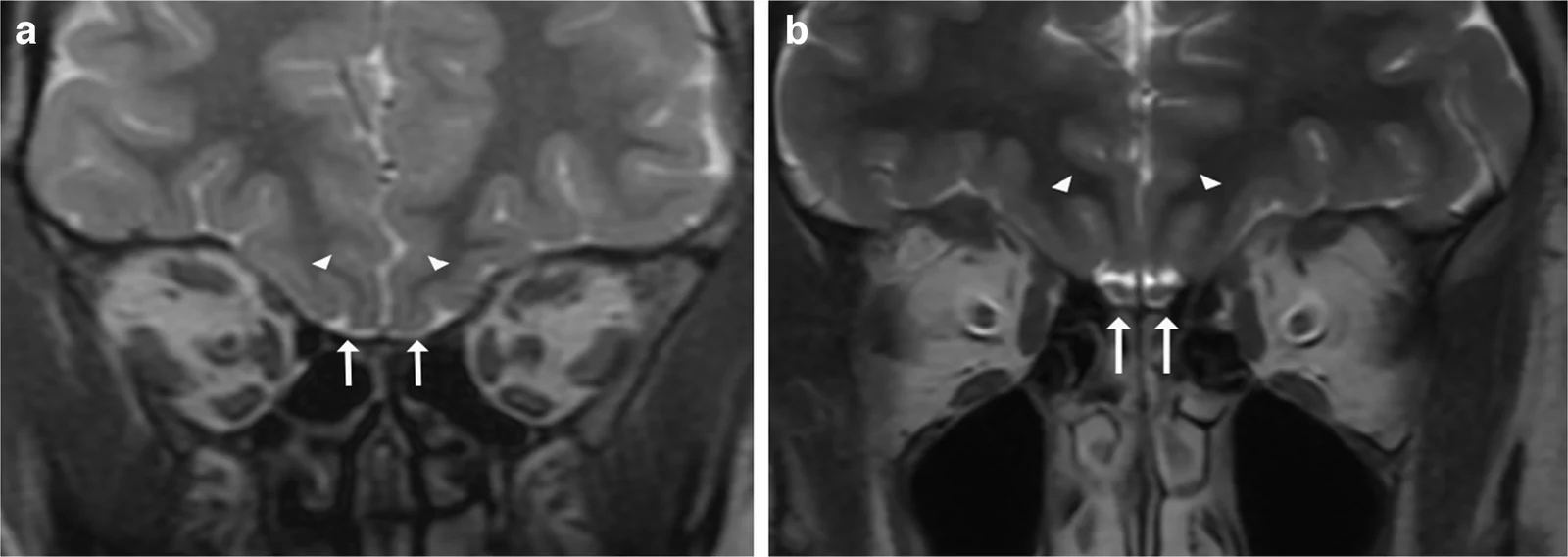
Fourteen-year-old male with anosmia and hypogonadotropic hypogonadism (Kallman syndrome). **a** Coronal T2-weighted sequence through the bifrontal region shows absence of the bilateral olfactory tracts in their expected locations (*arrows*). Shallow olfactory sulci are also noted (*arrowheads*). **b** For comparison, coronal T2-weighted image through the bifrontal region of a 15-year-old boy with the ability to smell shows a normal cross-sectional appearance of the bilateral parallel olfactory tracts (*arrows*) and deep olfactory sulci (*arrowheads*)

Another congenital diagnosis affecting pituitary function is Kallmann syndrome, a congenital form of hypogonadotropic hypogonadism caused by a deficiency in gonadotropic-releasing hormone (GnRH), often coupled with hyposmia or anosmia. This syndrome is estimated to affect approximately one in 10,000 males [22]. MR imaging of the olfactory sulci, best assessed in the coronal plane with T2-weighted imaging, may reveal the classic findings of agenesis or hypoplasia of the olfactory bulbs and olfactory sulci (Fig. 7). Although the majority of patients will have these structural findings, a small percentage may appear normal [22, 23]. Also noteworthy is the observation that most patients with this disorder have pituitary hypoplasia [23] but without additional hormone deficiencies. Additional laboratory and imaging evaluation is required to assess the extent of each individual’s GnRH deficiency [22]. Typically, these patients will have low gonadotropins, low testosterone levels (46,XY individuals), low estradiol levels (46,XX individuals), and low inhibin B levels. Given the possibility of familial inheritance in approximately 40% of cases, with X-linked, autosomal dominant and recessive patterns documented with Kallmann syndrome, a genetics consultation is also warranted for these patients [23]. The mainstay of treatment involves life-long sex hormone replacement therapy. Time-limited gonadotropin replacement therapy or pulsatile GnRH therapy can be considered for attempted induction of gonadal maturation and/or fertility induction.

## Developmental intracranial processes affecting hypothalamic-pituitary function: Hypothalamic hamartoma

**Fig. 8:**
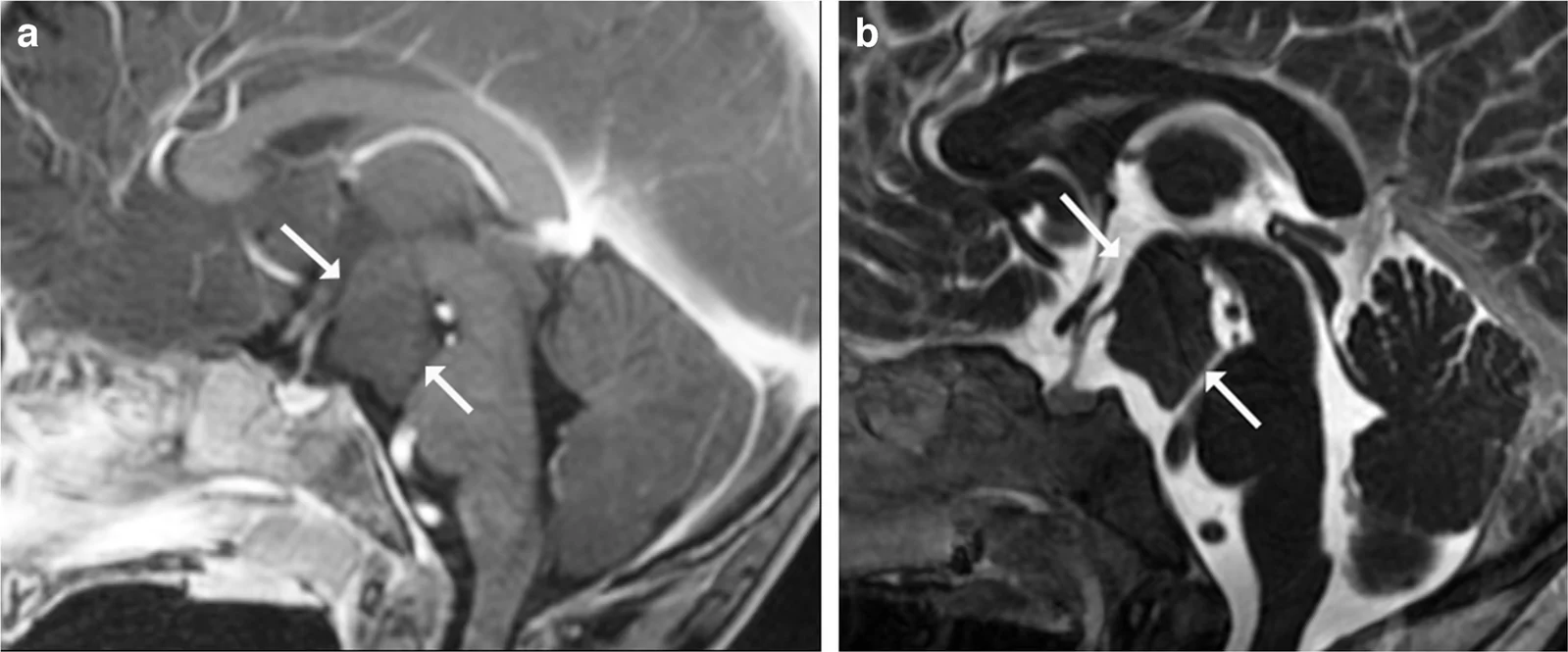
Three-year-old female with gelastic seizures and intrahypothalamic (sessile) hamartoma. **a ** Sagittal T1-weighted post-contrast and (**b**) sagittal volumetric T2-weighted steady-state acquisition images show a non-enhancing and uniformly isointense hamartoma (*arrows*) centered at the level of the hypothalamus

**Fig. 9:**
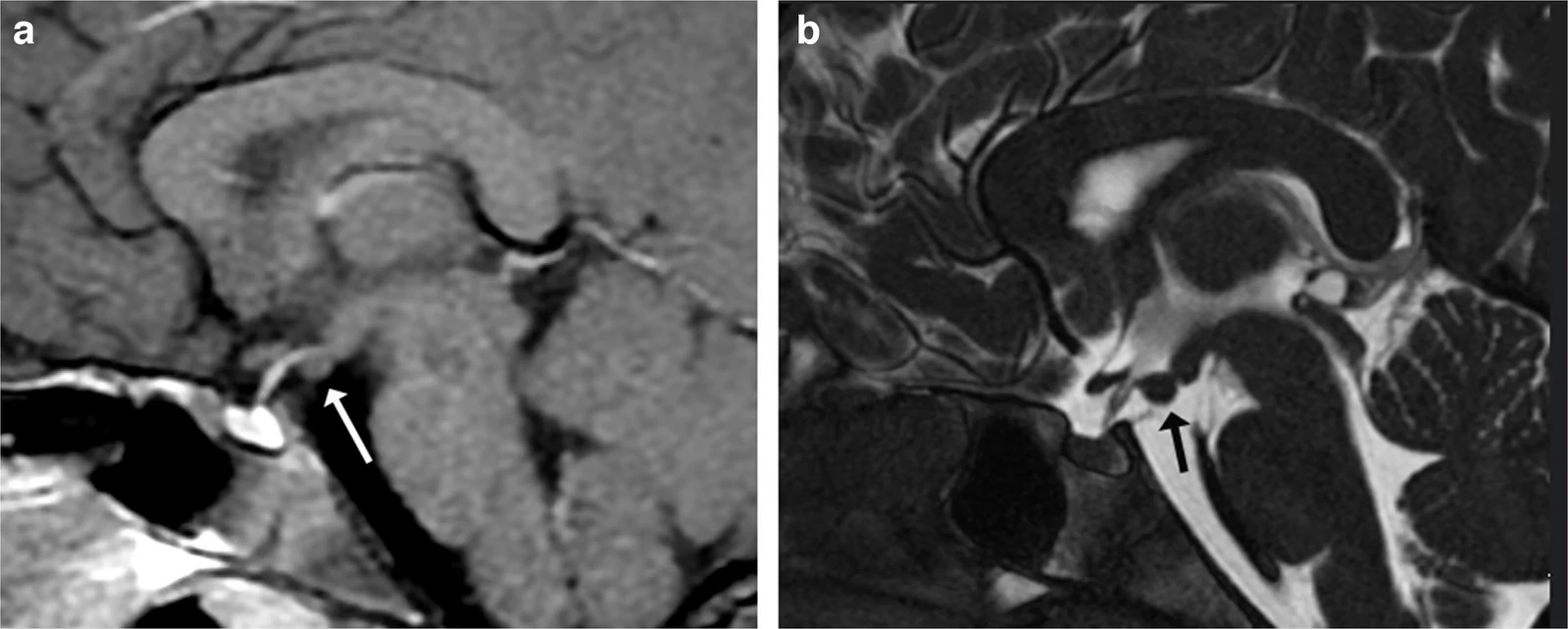
Six-year-old female with central precocious puberty and parahypothalamic (pedunculated) hamartoma. **a ** Sagittal T1-weighted post-contrast and (**b**) sagittal volumetric T2-weighted steady-state acquisition images show a non-enhancing and uniformly isointense nodule of tissue positioned tangential to the inferior surface of the hypothalamus (*white arrow* in **a**, *black arrow* in **b**)

Hypothalamic hamartomas are rare developmental, benign growths of abnormally organized neural tissue arising from the ventral aspect of the hypothalamus, at the tuber cinereum. Two different phenotypes of hypothalamic hamartomas have been described, with distinct clinical presentations and anatomic distributions: intrahypothalamic and parahypothalamic hamartomas [24]. The intrahypothalamic (sessile) hamartoma is positioned level to the mammillary bodies and third ventricle and is most strongly associated with gelastic seizures and other seizure types (Fig. 8). Endocrinopathies, namely central precocious puberty, and behavioral and developmental comorbidities may also occur [25]. In contrast, the parahypothalamic (pedunculated) hamartoma projects inferiorly from the ventral hypothalamus and is usually only associated with endocrinopathy, most commonly central precocious puberty (Fig. 9). Epilepsy and cognitive and behavioral disturbances can occur, although this is rare [26]. The central precocious puberty associated with a pedunculated hypothalamic hamartoma is attributed to a premature pulsatile release of hypothalamic gonadotropin-releasing hormone (GnRH) [25].

**Fig. 10:**
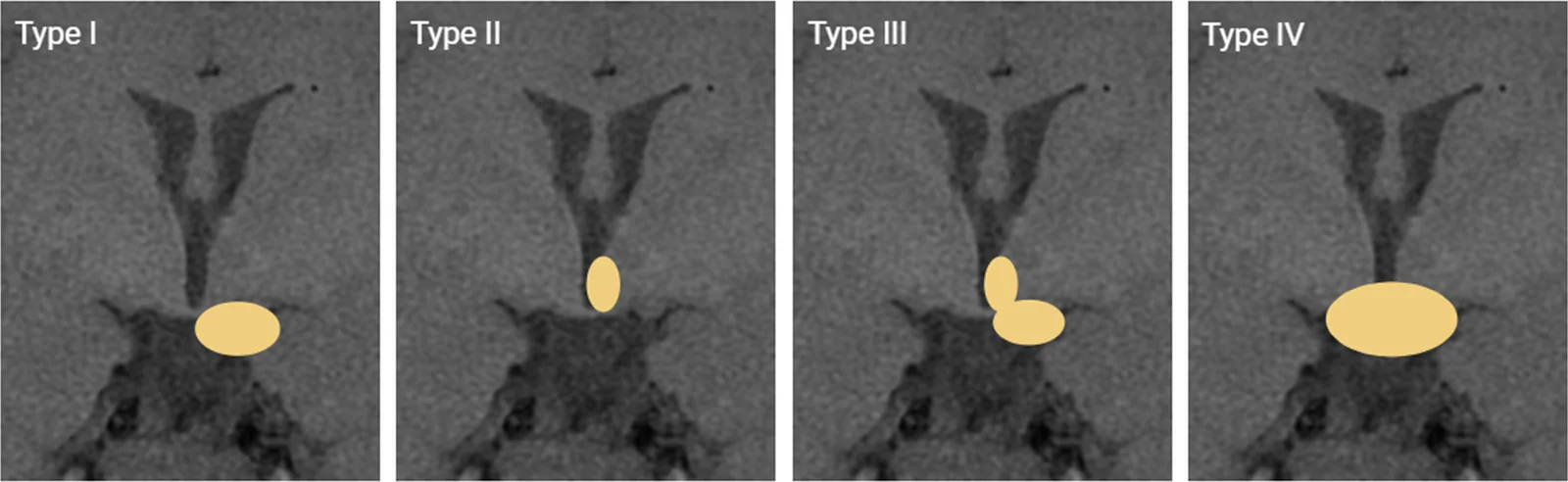
Delalande Classification of hypothalamic hamartomas. Schematic illustrations of the topographical classification of the hypothalamic hamartoma into four types, with optimal surgical disconnection routes, as described by Delalande et al. [27]. Type 1: subhypothalamic extension, horizontal insertion plane, pterional surgical approach. Type 2: ventricular extension, vertical insertion plane, endoscopic approach. Type 3: contains types 1 and 2 features, with the option for successive disconnection through the two approaches. Type 4: giant hamartoma of the hypothalamus, characteristics close to type 3, requiring a bilateral approach

The Delalande Classification is a system for categorizing hypothalamic hamartomas (HH) based on morphology observed on imaging or intraoperatively and may be used at some centers to influence the surgical approach and to predict outcomes [27]. Four types of HHs are defined in this classification system (Fig. 10): type I HH is attached to the hypothalamus with a narrow base (pedunculated), remaining extraventricular; type II HH is attached with a broad base to the floor of the third ventricle; type III HH is a straddling lesion that extends both into the third ventricle and the interpeduncular cistern; type IV HH is located entirely within the third ventricle (intraventricular). In single-center and multisite reviews, the HH type predicts both clinical presentation and surgical outcomes [28–30]. As mentioned above, the pedunculated (type I) lesions show the highest rate of precocious puberty, while type IV lesions demonstrate the highest frequency of gelastic seizures [28].

Treatment strategies include antiepileptic medications and medical suppression of puberty. Surgical resection is reserved for treatment of refractory epilepsy. Surgical outcomes vary significantly by type using the Delalande Classification. In a retrospective series of 214 patients with HH, type IV lesions achieve the best seizure freedom rates (91.7%), while type II lesions had the poorest outcomes (20% seizure-free) [28]. Overall, smaller, more laterally positioned lesions (types I and IV) respond better to minimally invasive approaches than larger straddling lesions [29, 30]. Central precocious puberty is effectively managed with GnRH agonist therapy, available as depot intramuscular injections administered every 3–6 months (i.e., leuprolide acetate) or as an implant replaced every 1–2 years (i.e., histrelin). Administration of GnRH in a non-pulsative manner inhibits the release of gonadotropins, effectively shutting off the hypothalamic-pituitary–gonadal axis responsible for central puberty. Treatment is successful at suppressing secondary sexual characteristics as well as preserving attainment of normal adult height and is typically continued until the child reaches typical pubertal age [31].

**Fig. 11:**
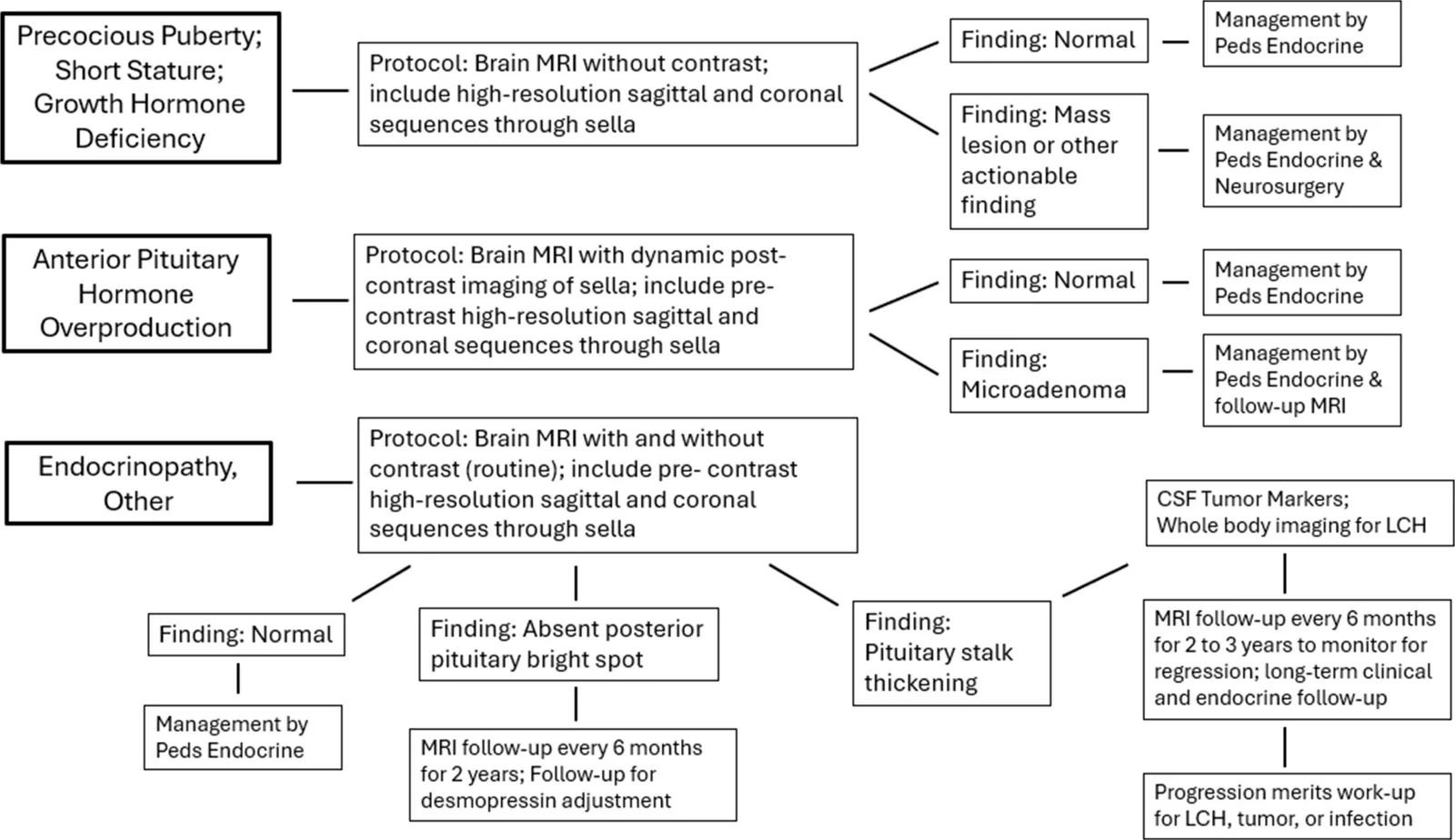
Brain magnetic resonance imaging protocol determination and subspecialty management based on clinical presentation and findings [2, 32]

Given that the etiologic considerations for central precocious puberty include hypothalamic hamartoma, diagnostic imaging using non-contrast brain MRI is appropriate, as hamartomas are non-vascular lesions that do not enhance and therefore do not require administration of gadolinium contrast agents for diagnosis [2]. However, the differential diagnosis for central precocious puberty, particularly for males, includes other intracranial lesions that could interrupt the normal puberty-inhibitory tracts, such as glioma (discussed below). Should additional clinical signs and symptoms raise suspicion of a tumor based on the neurologic or ophthalmologic exam, or based on a tumor predisposition syndrome, this would merit a contrast-enhanced brain MRI exam (see Fig. 11 for the imaging and management algorithm) [2].

**Fig. 12:**
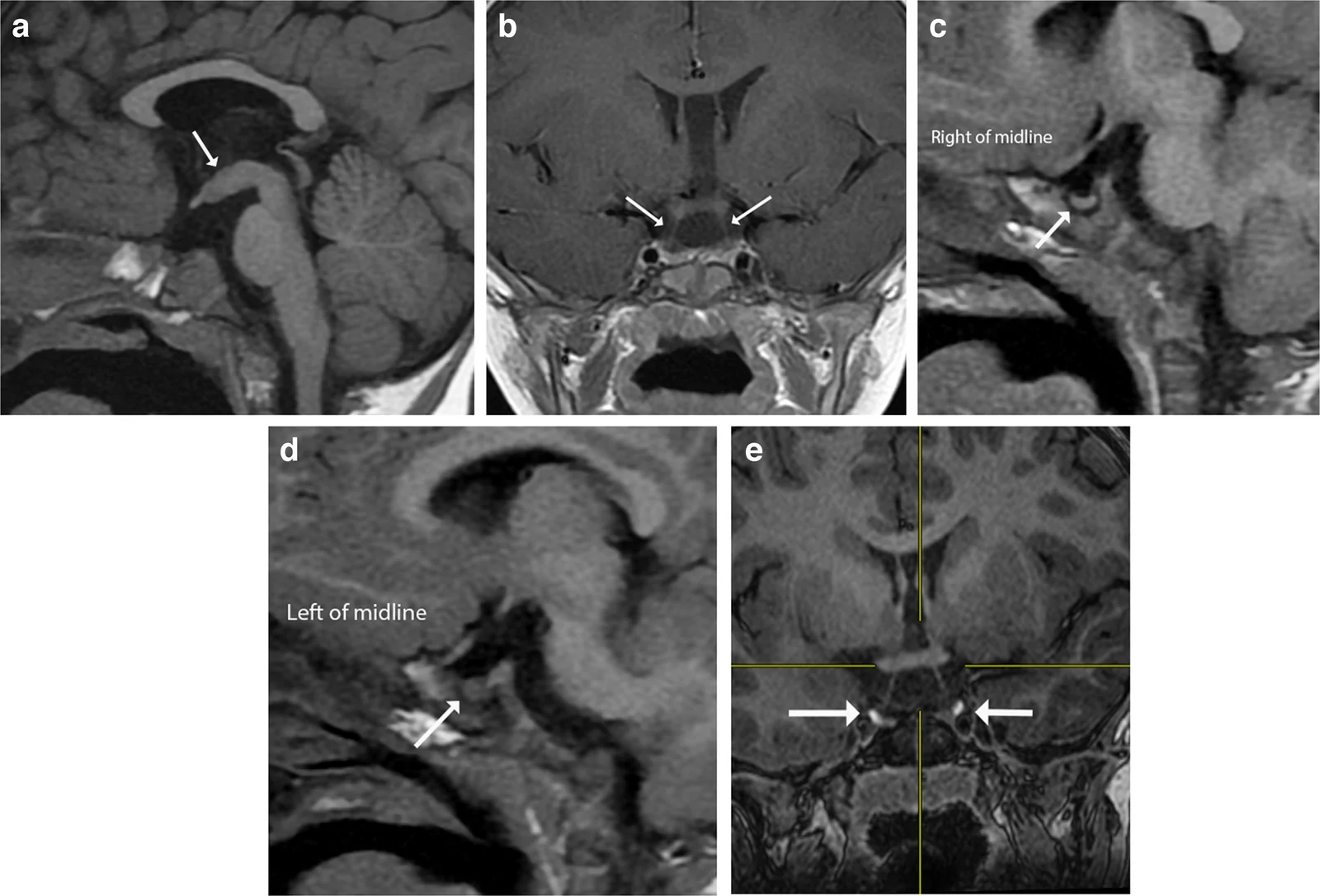
One-year-old female with short stature, originally presenting with a resected oral teratoma and head imaging unexpectedly identifying tubulomammillary fusion in the setting of a duplicated pituitary. **a** Sagittal T1-weighted sequence through the midline shows a uniform tubular thickening of tissue extending anteriorly from the midbrain along the hypothalamus in a manner that may mimic a hamartoma (*arrow*). **b** Coronal T1-weighted sequence through the sella shows duplicated, bilateral pituitary infundibula (*white arrows*, **b**). **c, d** Sagittal T1-weighted images through each hemi-sella show two distinct adenohypophyses (*arrows*). **e** Reconstructed coronal T1-weighted image through the hypothalamus, pituitary stalks, and bilateral pituitary bright spots (*arrows*, **e**). Laboratory studies followed over many subsequent years have been within normal ranges

An interesting imaging finding that may mimic a hypothalamic hamartoma is tuberomammillary fusion, which creates the appearance of tubular thickening across the hypothalamus region, earning the term, “hypothalamic pseudohamartoma” (Fig. 12). This anatomic abnormality has been reported to occur in 50–60% of duplicated pituitary gland cases, which are an extremely rare congenital malformation. Additional anomalies associated with duplicated pituitary gland include broadening or duplication of the sella (Fig. 12), broadening or duplication of the optic chiasm, duplication of the basilar artery, agenesis/hypoplasia of the corpus callosum, hypertelorism, cleft palate, supernumerary teeth, oropharyngeal teratomas, absent olfactory bulbs and/or tracts, cerebellar hypoplasia, and pontine hypoplasia [33]. Not surprisingly, endocrinopathies may occur, particularly central precocious puberty, requiring pubertal suppression therapy [34].

## Acquired intracranial processes affecting hypothalamic-pituitary function: Sella/suprasellar neoplasms

Suprasellar tumors can cause endocrine dysfunctions due to their close proximity to the pituitary gland and hypothalamus. These include hypopituitarism and central precocious puberty, as well as hypothalamic dysfunction, depending on the degree of compression and/or invasion of the hypothalamic-pituitary axis [35]. Diagnostic imaging reporting of a suprasellar mass on a brain MRI obtained for these endocrine presentations should indicate the presence or absence of obstructive hydrocephalus, evidence of metastatic spread through the cerebrospinal fluid spaces, and provide a reasonable differential diagnosis.

**Fig. 13:**
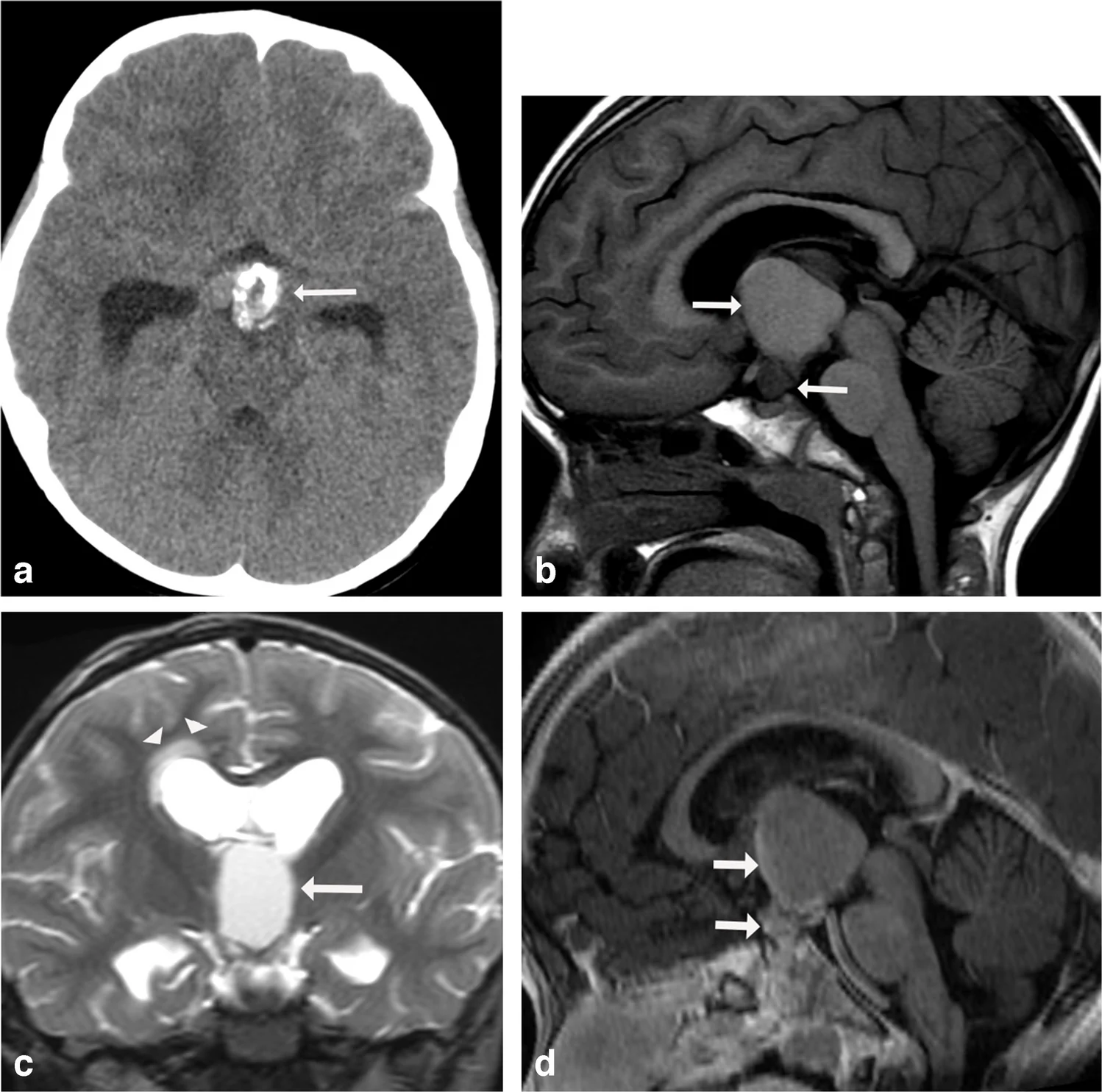
Three-year-old female presenting with polyuria, polydipsia, headache, and increasing somnolence, diagnosed with adamantinomatous craniopharyngioma. **a** Axial non-contrast computed tomography head at the level of the interpeduncular cistern shows a partially calcified mass in the suprasellar space (*arrow*). **b** Sagittal T1-weighted brain magnetic resonance imaging through the midline shows a large T1-hyperintense cystic mass arising from the sella and extending into the suprasellar space (*arrows*). **c** Coronal T2-weighted sequence shows a portion of the cystic mass (*arrow*) obstructing the foramen of Monro, with acute hydrocephalus and periventricular high T2-weighted signal indicative of transependymal edema (*arrowheads*). **d** Post-contrast sagittal T1-weighted sequence shows faint marginal enhancement around the dominant cystic component and minimal heterogeneous enhancement of the solid calcified portion (*arrows*)

**Fig. 14:**
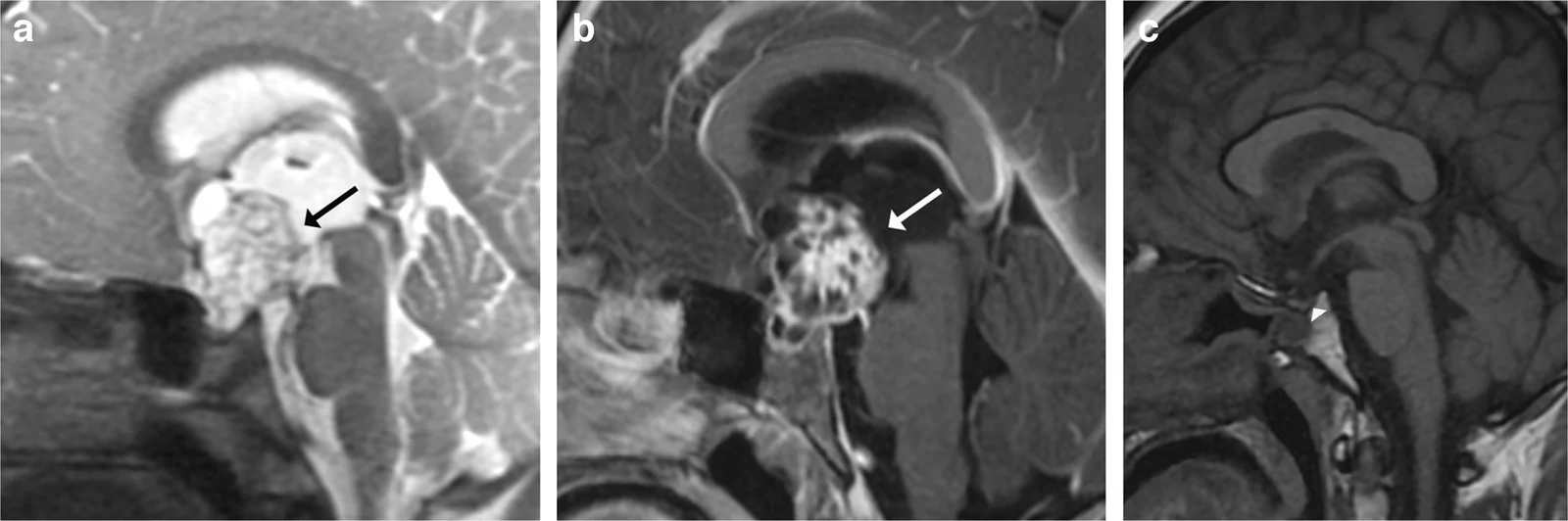
Fifteen-year-old male with treated adamantinomatous craniopharyngioma discovered at age 6 years for headache evaluation and multiple resulting endocrine abnormalities, including adrenal insufficiency, growth hormone deficiency, central hypothyroidism, gonadotropin deficiency, and diabetes insipidus. **a** Initial sagittal volumetric T2-weighted sequence and (**b**) sagittal post-contrast T1-weighted image through the midline show a mixed cystic and solid lobulated mass in the sella and suprasellar space (*arrows*). **c** Follow-up sagittal T1-weighted sequence shows interval resection of the tumor via a transsphenoidal approach. No discernible pituitary infundibulum. Thin pituitary tissue is identified in the sella (*arrowhead*)

The most commonly diagnosed suprasellar tumor in the pediatric population is the craniopharyngioma, a low-grade tumor [36]. There are two types of craniopharyngioma, each with distinctive clinical demographics, imaging features, histopathologic findings, genetic alterations, and methylation profiles. Adamantinomatous craniopharyngioma is the type seen most commonly in children, accounting for 5–11% of intracranial tumors in the pediatric population and characterized by *CTNNB1* alterations (*CTNNB1* encodes portions of the beta-catenin protein, involved in cell-to-cell adhesion and gene transcription) [37–39]. In contrast, the papillary craniopharyngioma occurs nearly exclusively in middle-aged adults and is driven by alterations in *BRAF* which impacts intracellular signaling and cellular proliferation [37, 38]. On brain MRI, a typical adamantinomatous craniopharyngioma appears as a large, predominantly cystic tumor with T1-hyperintense proteinaceous cyst contents (Fig. 13). The solid components may display contrast enhancement, and peripheral calcifications are present in the majority of adamantinomatous craniopharyngiomas [36, 40, 41]. In contrast, papillary craniopharyngiomas tend to be more solid and round, and less than 10% of papillary craniopharyngiomas calcify [40]. Of note, adamantinomatous craniopharyngiomas have been observed to more frequently invade the pituitary stalk, resulting in sacrifice of the pituitary stalk at the time of surgery as compared to the papillary craniopharyngiomas seen in adults. Accordingly, the rates of pituitary hormone deficiencies tend to be higher in children (Fig. 14) [41]. These tumors may cause obstructive hydrocephalus, requiring supratentorial shunting. Other clinical manifestations can include hypothalamic/pituitary deficiencies or visual impairment due to mass effect upon the optic chiasm.

**Fig. 15:**
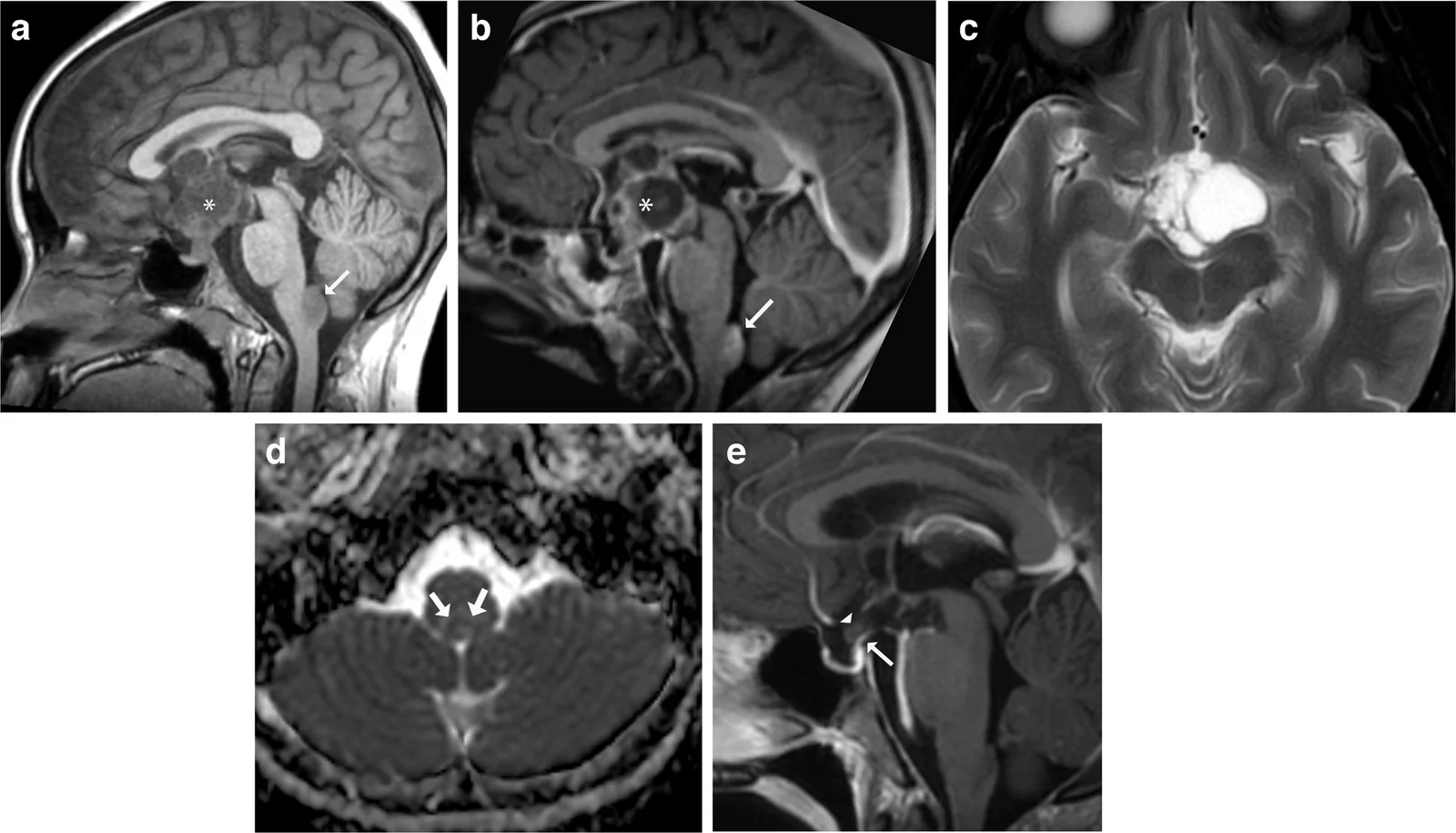
Suprasellar germinoma and metastatic focus in a 15-year-old female with primary amenorrhea and altered mental status at initial presentation. Tumor resection resulted in central diabetes insipidus, central hypothyroidism, and adrenal insufficiency. Sagittal (**a**) pre-contrast and (**b**) post-contrast T1-weighted image through the midline shows a large multilobulated, heterogeneous mixed cystic and solid mass with enhancement of solid components (*asterisk*) and hypointense ill-defined and faintly enhancing lesion at the cervicomedullary junction (*arrow*). Axial (**c**) T2-weighted image demonstrates multiple non-enhancing cystic components intermixed with enhancing solid tissue. **d** Axial ADC shows a rim of low-apparent diffusion coefficient (*arrows*, isointense to cerebellum) of the round metastatic focus at the obex. **e** Sagittal post-contrast T1-weighted image through the midline at 1-year follow-up surveillance exam shows post-resection findings. The sella has an empty appearance. Pituitary infundibulum is visualized (*arrow*), and enhancing tissue along floor of sella suggests a thin rim of residual adenohypophysis. Residual nodule of non-enhancing tissue (*arrowhead*) at the level of the hypothalamus remained stable over subsequent exams (not shown)

**Fig. 16:**
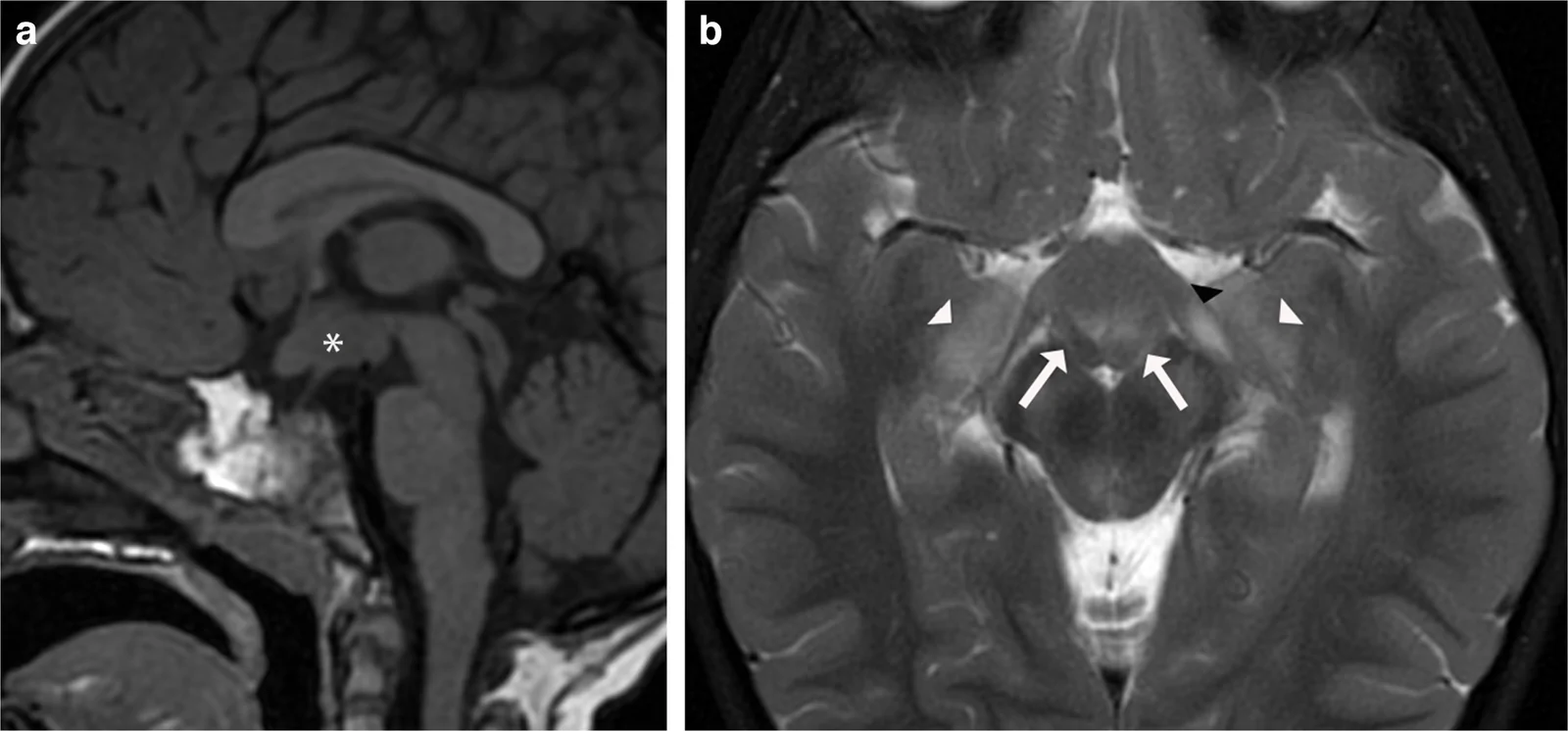
Optic pathway glioma in a 3-year-old female with a known history of neurofibromatosis type 1 presenting with growth hormone excess. **a** Sagittal T1-weighted image through the midline shows a T1-isointense 2-cm mass (*asterisk*) centered at the optic chiasm. **b **Axial T2-weighted image shows the lesion (*arrows*) centered at the level of the hypothalamus between the optic tracts and the cerebral peduncles. Hyperintense T2-weighted signal is partially visualized in the left optic tract (*black arrowhead*), consistent with involvement by optic pathway tumor, and bilateral hippocampal formations (*white arrowheads*), reflecting the myelin vacuolar changes typical of neurofibromatosis type 1

The differential diagnosis for a mixed solid and cystic tumor in the suprasellar space in a child includes a germinoma, a type of germ cell tumor of the CNS. Intracranial germinoma is a rare malignant tumor that is usually diagnosed in children and young adults. The majority of these tumors arise in the pineal region, but approximately one-third of recorded cases arise in the suprasellar space [42, 43]. Suprasellar germinomas tend to be more solid than craniopharyngiomas and can sometimes be distinguished by their hypercellularity in solid regions, based on restricted diffusion using diffusion-weighted imaging. Because of its higher histological grade, this tumor type may have ill-defined margins with invasion of the brain parenchyma or may present with seeding of tumor through the cerebrospinal fluid spaces (Fig. 15). Other pediatric intracranial tumors that occur in the suprasellar space include pilocytic and pilomyxoid astrocytomas. In the suprasellar location, these tumor types appear round, with well-defined margins and often uniform enhancement [44]. Neurofibromatosis type 1 (NF1)-associated pilocytic astrocytomas typically involve the optic pathway and appear solid rather than cystic [45]. Interestingly, growth hormone excess resulting in an unexpected upward growth trajectory has been observed to occur in children with NF1 and optic gliomas, even without central precocious puberty (Fig. 16) [46]. Tissue sampling of any identified suprasellar mass outside the context of known NF1 is required for histological and genetic alteration testing to guide management [44].

It is imperative that pituitary hormone deficiencies – especially cortisol deficiency - are screened for and addressed prior to tumor resection, as they can result in severe perioperative complications if untreated. There is a high likelihood for postoperative diabetes insipidus, sometimes accompanied by a triphasic response of subsequent SIADH followed by permanent diabetes insipidus. Overall, endocrine function after removal of these tumors is notoriously poor with high rates of both posterior and anterior pituitary dysfunction (Figs. 14 and 15) [36]. Additionally, these patients have a high incidence of increased weight gain post-operatively, which is likely due to hypothalamic damage leading to derangements in energy balance and satiety.

## Acquired intracranial processes affecting hypothalamic-pituitary function: Pituitary adenomas

Pituitary adenomas are non-malignant tumors estimated to be present in nearly one-quarter of the population [47]. These adenomas may be non-functioning or functioning, with overproduction and secretion of pituitary hormones. In the pediatric population, the incidence of pituitary adenomas is much higher in females than males (75–90% of identified adenomas occur in girls) [47, 48]. If large enough, mass effect created by an adenoma may lead to endocrine dysfunction or visual disturbances. Pituitary adenomas are differentiated as micro- or macroadenomas by their size classification and location, with microadenomas falling within the bounds of the anterior pituitary and measuring less than 10 mm [49, 50].

**Fig. 17:**
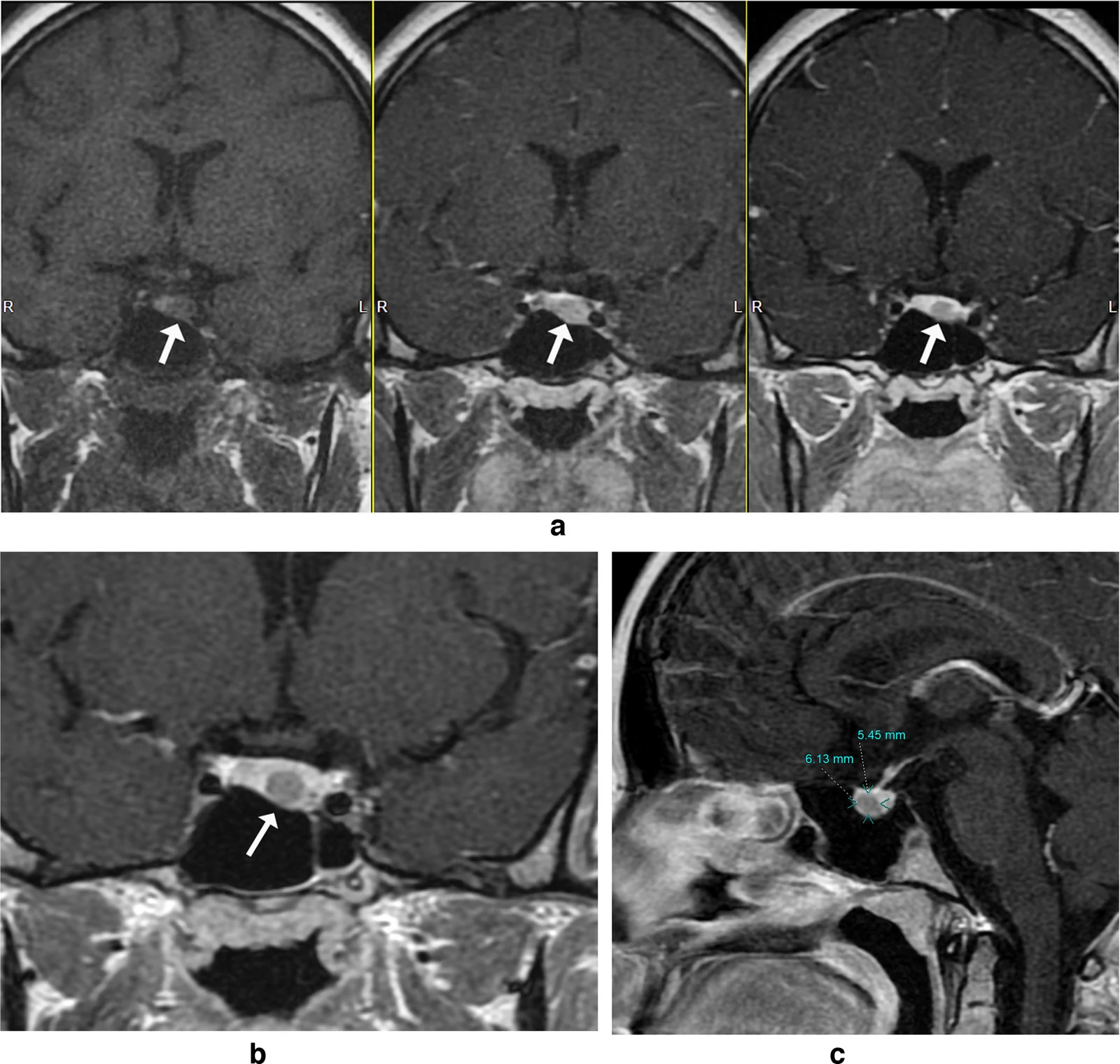
Microadenoma in a 15-year-old female with elevated prolactin levels. **a** Coronal dynamic post-contrast T1-weighted images through the sella show progressive enhancement of the adenohypophysis, delineating a non-enhancing round region (*arrows*), consistent with microadenoma. **b** Coronal and (**c**) sagittal delayed post-contrast images through the pituitary show the well-demarcated non-enhancing lesion (*arrow* in **b**) that measures 6 mm in diameter, confirming the presence of a prolactinoma

Clinical suspicion for a hormone-producing microadenoma merits brain MRI using a high-resolution pituitary protocol, and both non-contrast and gadolinium contrast-enhanced brain MRI exams are listed as usually appropriate in current American College of Radiology Appropriateness Criteria [51]. Dynamic contrast-enhanced MRI takes advantage of the delayed enhancement of a microadenoma relative to the normal anterior pituitary gland [52, 53]. The anterior pituitary gland receives its blood supply from the superior hypophyseal arteries indirectly through the pituitary portal system [54]. Using dynamic contrast-enhanced T1-weighted spin-echo MR imaging of the pituitary gland in the coronal plane with a temporal resolution of 12 s to 20 s, a gradual enhancement of the gland can be observed, often from the central portion of the tissue in an outward radiating fashion, although the enhancement pattern is variable. Pituitary microadenomas are characterized by a delayed enhancement such that they stand out as non-enhancing T1-isointense round 3–10-mm lesions against a background of enhancing anterior pituitary over a scanning period of 1 min to 3 min (Fig. 17). A dynamic contrast-enhanced MRI protocol for pituitary microadenoma identification is summarized in Table 1.

**Table 1:** MRI parameters for focused pituitary imaging with non-contrast T1- and T2-weighted sequences and dynamic contrast-enhanced (*DCE*) MRI, performed for suspected microadenoma

Sequence	TR/TE (ms)	Slice thickness (mm)	FOV (mm)	Matrix size	Resolution (mm^2^)
Sag T1W FSE	650–800/12	3.0	180	256×192	0.8×1.0
Sag T2W FSE with fat-sat	5,500/90	3.0	180	256×192	0.8×1.0
Cor T1W DCE-MRI (6 phases; each 6–7 slices through pituitary)	700–800/10	2.0	160	256×192	0.8×1.0

Comprehensive whole-brain sequences are run in addition to focused pituitary imaging of sella/suprasellar region

*Cor* coronal,* FSE* fast spin echo, *FOV* field of view, *Sag* sagittal, *T1W* T1-weighted,* T2W* T2-weighted, *TE* time of echo, *TR* time of repetition

While dynamic contrast-enhanced MRI constitutes the current gold standard in the diagnosis of pituitary microadenoma, the known gadolinium retention and other potential risks of gadolinium administration warrant consideration of relying on non-contrast imaging [2, 55–57]. Although some microadenomas can be identified by non-contrast T1-weighted and T2-weighted sequences, with the sensitivity of non-contrast imaging for detection of microadenomas ranging from 56–71%, the addition of dynamic contrast-enhanced techniques increases sensitivity to 80–87% [53, 58]. However, an important consistent observation is that microadenomas show minimal to no increase in size on follow-up imaging [53], supporting the reliance upon non-contrast brain MRI for follow-up exams or eliminating routine follow-up brain imaging entirely, at least for non-secreting microadenomas [53, 59].

**Fig. 18:**
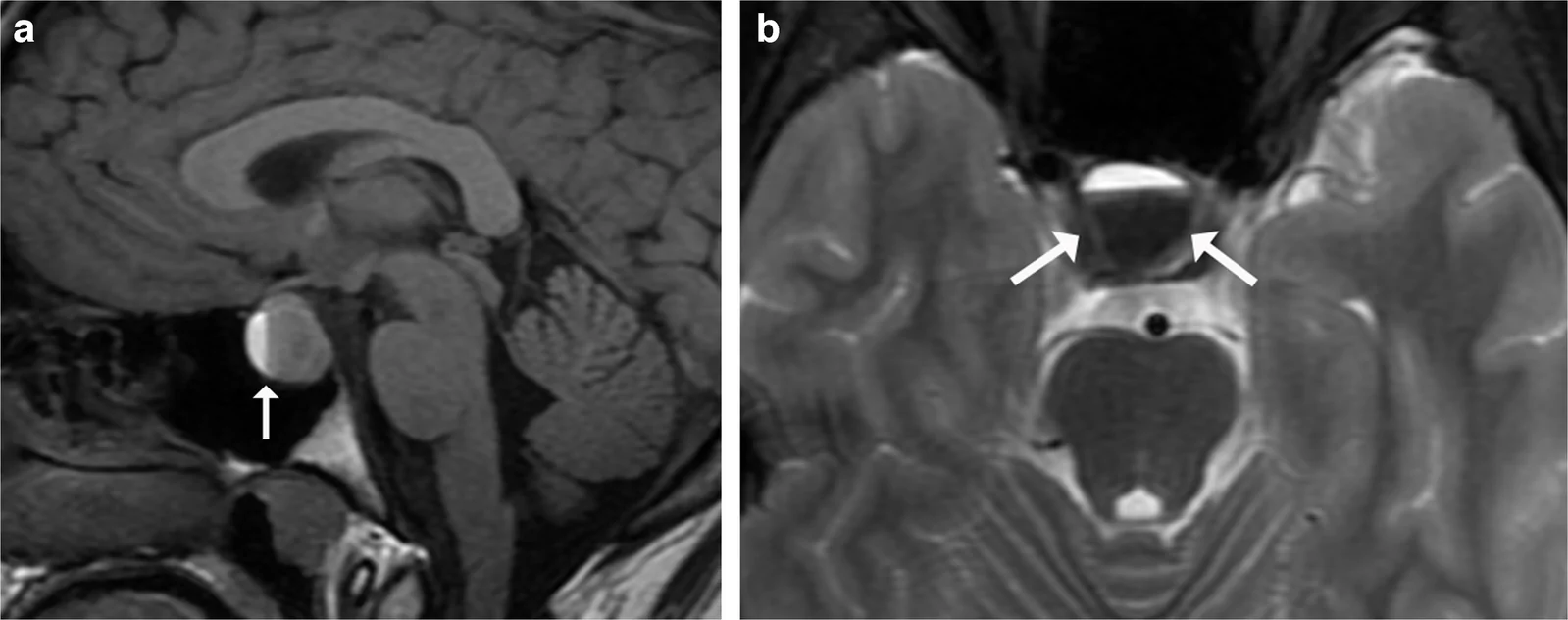
Macroadenoma in a 16-year-old female with elevated prolactin levels. **a **Sagittal T1-weighted sequence through the midline shows a rounded lesion in the sella containing a heme-fluid level (*arrow*) measuring 18 mm in diameter. **b **Axial T2-weighted image through the sella redemonstrates the lesion with a heme-fluid level

Pituitary macroadenomas are distinct from microadenomas in several ways. As mentioned above, macroadenomas are larger than 10 mm. Whereas microadenomas commonly occur in the pediatric population, macroadenomas predominantly occur in middle-aged adults, although these tumors are uncommonly diagnosed in adolescents [52]. Because most of these lesions are non-functioning, affected individuals most frequently present with headache and/or vision loss due to mass effect by the macroadenoma. Finally, whereas microadenomas appear homogeneous in signal and enhancement on MRI, macroadenomas are frequently heterogeneous in signal. They may contain cystic components and hemorrhage (Fig. 18), and they present with a wide range of volumes, sometimes with extension beyond the cavernous sinuses [52].

**Fig. 19:**
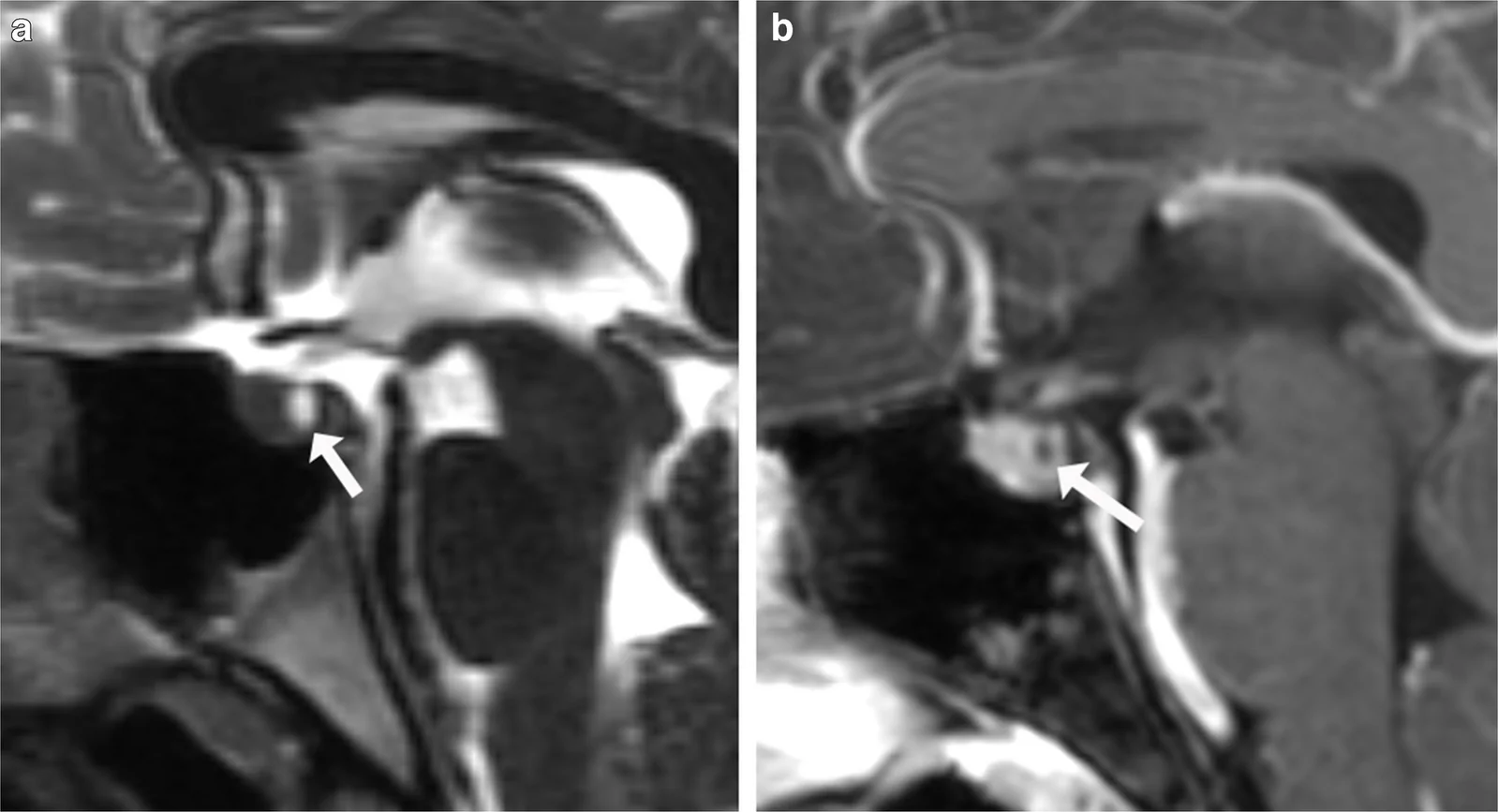
Rathke cleft cyst incidentally identified on a brain magnetic resonance imaging performed on a 16-year-old female with headache. **a** Sagittal T2-weighted image and (**b**) sagittal post-contrast T1-weighted image show a small rounded region of hyperintense T2-weighted signal (*arrow*) and non-enhancing isointense T1-weighted signal (*arrow*) located between the anterior pituitary and the posterior pituitary bright spot, consistent with a small Rathke cleft cyst

**Fig. 20:**
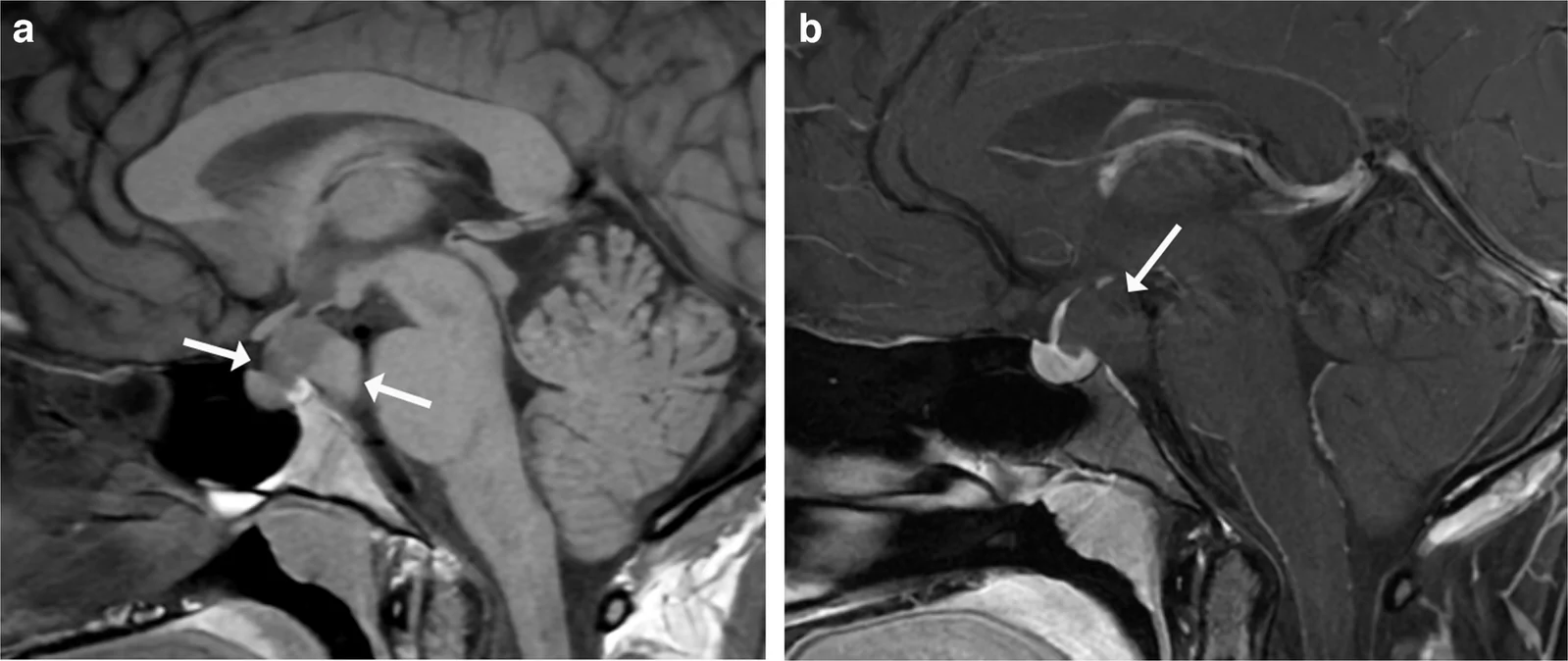
Rathke cleft cyst in an 18-year-old female with diplopia and elevated prolactin levels. Sagittal T1-weighted images acquired (**a**) pre-contrast and (**b**) post-contrast show a large, exophytic cystic lesion with a fluid-fluid level (*arrows* in **a**) hanging over the dorsum sella. Post-contrast image accentuates the infundibulum, displaced anteriorly by the mass effect of the cyst (*arrow* in **b**)

Distinct from a pituitary macroadenoma is a Rathke cleft cyst, which is typically small and confined to the sella, positioned between the anterior and posterior pituitary lobes (hence, also called a pars intermedia cyst) [60]. On imaging, a small Rathke cleft cyst is non-enhancing, with hypointense T1-weighted and hyperintense T2-weighted signal in the expected location (Fig. 19). However, some Rathke cleft cysts may be predominantly exophytic (Fig. 20). These benign lesions do not infiltrate the normal pituitary tissue, although if large enough, there may be mass effect evident upon the anterior pituitary. The most common associated endocrinopathies include hyperprolactinemia and hypogonadotropic hypogonadism, but secondary adrenal insufficiency, secondary hypothyroidism, and growth hormone deficiency have also been reported. Development of hormonal disruptions is more likely due to inflammation than solely due to mass effect [61]. Symptomatic cysts require intervention by transsphenoidal surgery for decompression of the cyst, although cyst recurrence is reported in up to a third of cases [60]. Minimally invasive endoscopic techniques have improved surgical outcomes, but postoperative complications including hypopituitarism and cerebrospinal fluid leaks still occur [60].

Pituitary adenomas may arise from any of the five hormone-producing cells in the anterior pituitary including somatotrophs, lactotrophs, corticotrophs, gonadotrophs, and thyrotrophs, each leading to a distinct clinical presentation related to the hormone being over-produced. Once a pituitary adenoma is identified on imaging, complete pituitary screening should be completed and managed accordingly [62]. Medical management may include dopamine agonists in the case of prolactinoma, metyrapone or ketoconazole in the case of Cushing disease (while awaiting definitive surgery), and somatostatin analogues in the case of GH excess or TSHoma [63]. Transsphenoidal surgery is the definitive treatment of choice for most pituitary adenomas and can often be curative but carries the risk of deranged vasopressin secretion post-operatively [62, 63]. Some pituitary adenomas may be treated with radiotherapy if the tumor is symptomatic, growing, resistant to medical therapy, and surgically inaccessible. There is mixed evidence of biochemical remission after radiotherapy, but these patients are at risk for developing hypopituitarism over time, requiring life-long pituitary hormone monitoring [62].

## Acquired intracranial processes affecting hypothalamic-pituitary function: Infiltrative diseases of hypothalamic-pituitary infundibulum

As described above, vasopressin (antidiuretic hormone) is normally transported from the hypothalamus through the neural component of the pituitary stalk and stored in nerve terminals within the posterior pituitary. Central or neurogenic diabetes insipidus (DI) refers to a deficiency in either the synthesis or secretion of vasopressin [9, 32]. The deficiency results in polydipsia and polyuria (high urine output and dilute urine). Central DI is a rare disease, with one European study reporting an annual incidence of 3–4/100,000 new cases and a higher prevalence in children and older adults above 80 years [64]. Causes for central DI include defects in the function of the hypothalamic osmoreceptors or defects in the nuclei of the hypothalamus, and median eminence of the hypothalamus, pituitary infundibulum, or the posterior pituitary gland [9]. Of the intracranial tumors discussed earlier, germinomas can cause central DI by damaging the hypothalamus or pituitary stalk, which are responsible for vasopressin production and release. While DI can be an isolated initial symptom, other signs of a germinoma include visual disturbances and signs of increased intracranial pressure.

**Fig. 21:**
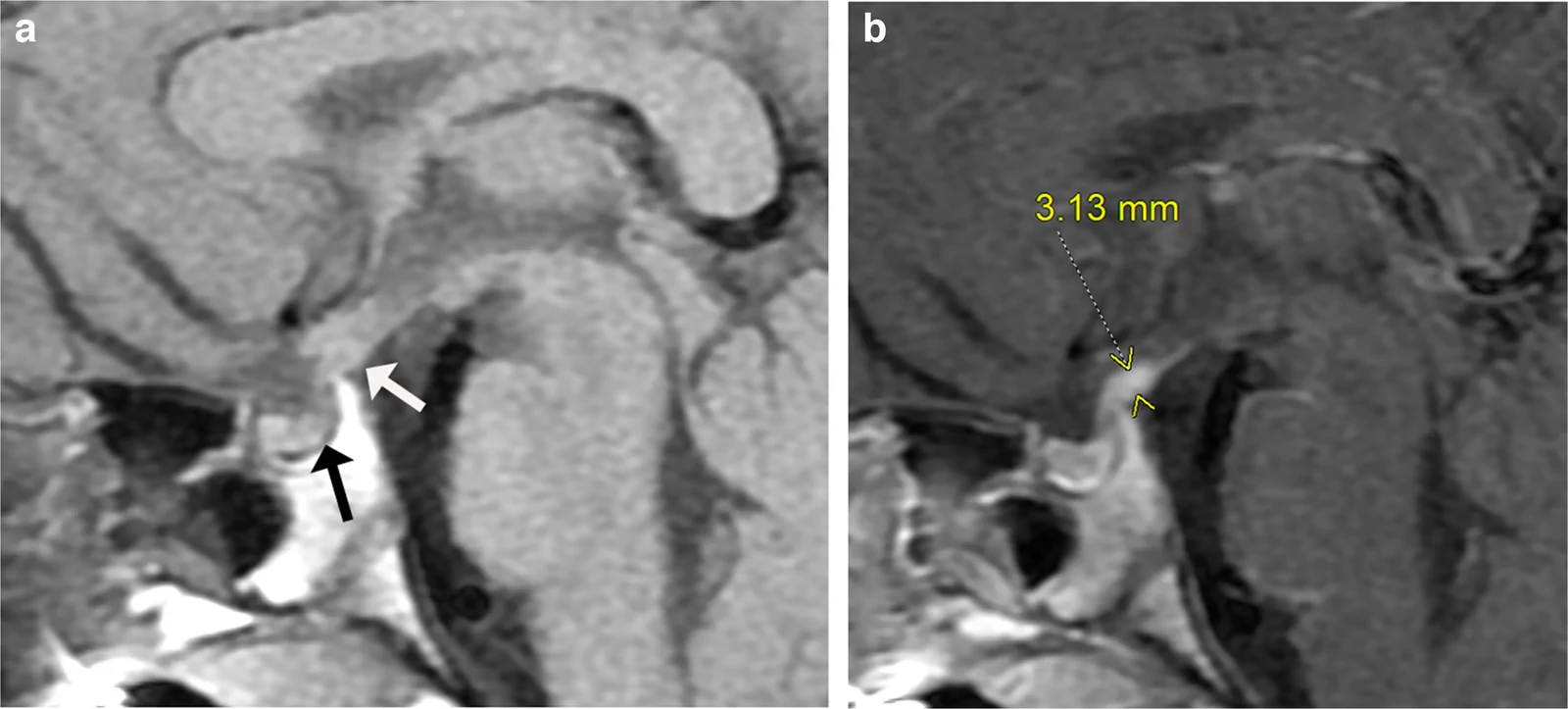
Ten-year-old male with central diabetes insipidus. **a** Sagittal T1-weighted brain MRI shows absence of the posterior pituitary bright spot (*black arrow* in **a**); no ectopic bright spot was present, but the isointense pituitary infundibulum appears thickened (*white arrow* in **a**). **b** Post-contrast image shows 3.1-mm thickening of the upper pituitary infundibulum (*calipers* in **b**). Differential considerations include infundibular-neurohypophysitis, IgG4 hypophysitis, Langerhans cell histiocytosis, and early manifestation of germ cell tumor. Tumor markers and cerebrospinal fluid assessment were normal. Etiology was presumed inflammatory. The patient was managed conservatively with repeat brain MRI exams over the following 2 years to exclude development of neoplasm (not shown)

In addition to the congenital and neoplastic entities discussed above, autoimmune inflammatory disorders of the neurohypophysis may occur, leading to infiltration of the pituitary infundibulum and pituitary gland by lymphocytes. The most recognized scenario for development of autoimmune hypophysitis is in pregnant or post-partum females [9]. However, autoimmune hypophysitis and autoimmune infundibular-neurohypophysitis can rarely occur in children, possibly mediated by IgG4 autoantibodies [65, 66]. MRI findings can include abnormal enlargement of the anterior pituitary gland, loss of the posterior pituitary bright spot, or thickening of the pituitary infundibulum (Fig. 21) [9, 32, 67]. MRI may establish the diagnosis without the need for a pituitary biopsy, although surgical transsphenoidal biopsy may be considered if there is diagnostic uncertainty [9].

**Fig. 22:**
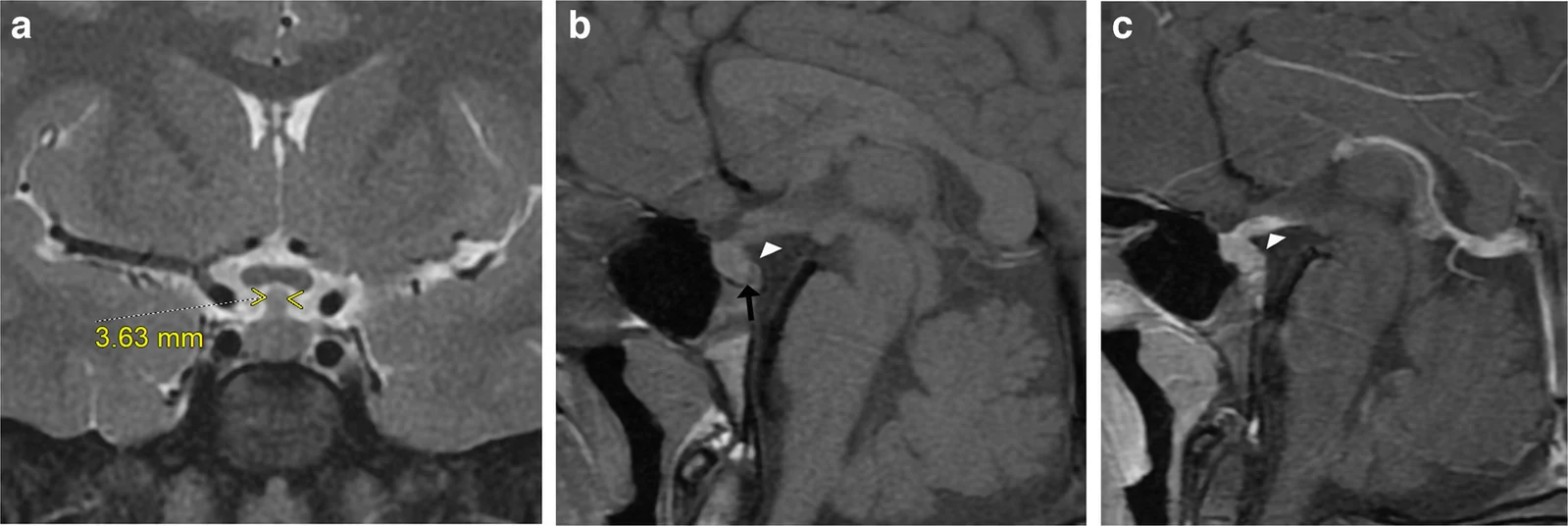
Eight-year-old female with central diabetes insipidus that resolved following therapy for presumptive Langerhans cell histiocytosis. **a** Coronal T2-weighted sequence through the sella shows abnormally thickened pituitary infundibulum, measuring 3.6 mm. Sagittal (**b**) pre-contrast and (**c**) post-contrast T1-weighted images of the sella redemonstrate abnormal infundibular thickening and enhancement to the level of the tuber cinereum (*white arrowheads*). Note also the absence of the posterior pituitary bright spot (*black arrow* in **b**). Follow-up imaging exams after a chemotherapy course show normalization of the infundibulum and no intrasellar mass (not shown)

Another diagnostic consideration for central DI in children is Langerhans cell histiocytosis (LCH), a hematological disorder with an array of forms ranging from solitary to disseminated and multisystemic [68]. When LCH localizes to the hypothalamus-pituitary region, it results in destruction of nervous tissue. Brain MRI findings include pituitary infundibulum thickening and/or an absent posterior pituitary bright spot (Fig. 22) [9, 67, 68]. The pituitary infundibulum is typically 1–2 mm in thickness, and an infundibulum greater than 3 mm proximally or greater than 2 mm distally should be considered abnormal. However, it is important to emphasize that neither absence of the posterior pituitary bright spot nor thickening of the pituitary infundibulum is specific for a particular disorder, discussed further below [32]. When LCH is a possible consideration for central DI in the setting of these findings, consultation by pediatric hematology-oncology is recommended to guide further evaluation and management. Additional diagnostic imaging may include a radiographic skeletal survey, whole-body ^18^fluorodeoxyglucose (FDG) PET-CT, or whole-body MRI screening exam to evaluate for disseminated disease [69–71]. The nonspecific findings of pituitary infundibular thickening or loss of the posterior pituitary bright spot may be seen with LCH, and also as an early manifestation of a germ cell tumor, lymphocytic infundibulo-hypophysitis, and other inflammatory or autoimmune conditions. For these reasons, identification of these findings requires long-term follow-up [32].

Additional known causes of central DI include head injury, genetic causes of defects in vasopressin synthesis, and diabetes insipidus, diabetes mellitus, optic atrophy, and deafness (DIDMOAD) syndrome, also known as Wolfram syndrome [9, 32]. In a minority of pediatric cases, an underlying cause of central diabetes insipidus cannot be established [72]. A diagnosis of idiopathic central diabetes insipidus is a diagnosis of exclusion and only can be made after extensive investigation and when serial MRI exams performed every 6 months over 2 years to 3 years fail to demonstrate a cause such as LCH, germinoma, or infection/inflammation. Idiopathic central DI is presumably caused by an autoimmune process [9, 32, 73].

Figure 11 provides a diagnostic imaging algorithm in the setting of central endocrinopathies depending on brain MRI findings. The congenital and acquired disorders reviewed here are listed in Table 2, with a summary of the endocrine abnormalities requiring evaluation and medical management.

**Table 2:** Pediatric intracranial disorders that cause endocrine abnormalities and their key imaging features

Intracranial pathology	Key features on brain imaging	Endocrine abnormalities and key clinical features
Congenital anomalies		
Ectopic posterior pituitary	T1-hyperintense posterior pituitary bright spot at the median eminence of hypothalamus or along pituitary stalk	• Isolated growth hormone deficiency • Combined pituitary hormone deficiencies • Pituitary function may remain normal
Partial ectopic posterior pituitary	Two distinct T1-hyperintense posterior pituitary bright spots, one in orthotopic position within the sella and one in ectopic position at median eminence of hypothalamus or along pituitary stalk	
Pituitary stalk interruption syndrome	• Thin, interrupted, or absent pituitary stalk • Hypoplasia of adenohypophysis • Ectopic posterior pituitary bright spot	
Duplicated pituitary gland	• Duplication of sella • Broadening of optic chiasm • Tuberomammillary fusion (hypothalamic pseudohamartoma) • Associated anomalies that may be present: duplication of basilar artery, pontine hypoplasia, dysgenesis of corpus callosum, hypoplastic olfactory tracts, hypertelorism, cleft palate, oropharyngeal teratomas, supernumerary teeth	
Hypogonadotropic hypogonadism (Kallman syndrome)	• Agenesis or hypoplasia of olfactory bulbs • Shallow or absent olfactory sulci • Anterior pituitary hypoplasia may be present	• Deficiency in gonadotropic-releasing hormone (GnRH) – low gonadotropins, low levels of testosterone in males, low estradiol in females, low inhibin B levels • Hyposmia or anosmia
Hypothalamic hamartoma		
Hypothalamic hamartoma – intrahypothalamic (sessile)	Isointense, non-enhancing mass lesion positioned level to the third ventricle and mamillary bodies	• Gelastic seizures and other seizure types • Precocious puberty may occur
Hypothalamic hamartoma – parahypothalamic (pedunculated)	Isointense, non-enhancing mass lesion that projects inferior to the hypothalamus	Typically, only associated with precocious puberty
Acquired mass lesions in sella/suprasellar space		
Intrasellar/suprasellar neoplasms	Craniopharyngioma • Combination of cystic and solid components • Calcifications are commonly present Germinoma • Tend to be mostly solid tissue but may have cystic components • Solid tissue is hypercellular and shows restricted diffusion • Ill-defined margins Pilocytic astrocytoma • Round solid mass with well-defined margins • Solid tissue is hypercellular and shows restricted diffusion Optic pathway glioma • Associated with NF1, a cancer predisposition syndrome • Variable involvement of the optic nerves, optic chiasm, optic tracts, lenticular thalamic nuclei, and optic	• Mass effects by tumors: headache, obstructive hydrocephalus, and visual disturbances due to impingement on optic chiasm Endocrine disorders: • Pan-hypopituitarism and variable degrees of hypothalamic dysfunction • Diabetes insipidus – particularly with germinomas, as these tumors originate in the posterior pituitary or infundibulum • Precocious puberty – may occur with germ cell tumors producing hCG • Growth hormone excess – reported to uncommonly occur in NF1 patients with optic pathway glioma Post-resection disorders: • Diabetes insipidus • High rates of anterior and posterior pituitary dysfunction
Pituitary microadenoma	3–10-mm round lesion within the adenohypophysis characterized by delayed enhancement	• Non-functioning microadenomas will be clinically silent, possibly incidentally observed on brain MRI acquired for other reasons • Functioning microadenomas: overproduction of ACTH, GH, LH, FSH, TSH, or prolactin
Pituitary macroadenoma	Lesion within the adenohypophysis that is larger than 10 mm, heterogeneous in signal, frequently with cystic and hemorrhagic components. May extend beyond the confines of the sella	• Non-functioning, benign • Headache • Vision loss/visual disturbances • Transsphenoidal surgery may result in derangements in ADH secretion • Radiotherapy for surgically inaccessible macroadenomas may develop pan-hypopituitarism over time
Rathke’s cleft cyst	Hypointense T1- and hyperintense T2-weighted small lesion without enhancement, positioned between the adenohypophysis and neurohypophysis; may be exophytic and extend above the height of the adenohypophysis	• Non-functioning, benign • If large enough, may have mass effect upon adenohypophysis or upon optic chiasm, resulting in visual disturbances
Acquired pituitary infiltrative lesions		
Pituitary involvement by Langerhans cell histiocytosis	Absence of posterior pituitary bright spot, with or without pituitary infundibular thickening; rarely hypothalamic mass/thickening may be observed	• Central diabetes insipidus is the most common endocrine manifestation • Other hypothalamic-pituitary axis deficiencies may develop
Autoimmune infundibular-neurohypophysitis	Abnormal enlargement of the anterior pituitary gland, loss of the posterior pituitary bright spot, or thickening of the pituitary infundibulum	• Central diabetes insipidus is the most common endocrine manifestation • Other hypothalamic-pituitary axis deficiencies may develop

*ACTH* adrenocorticotropic hormone, *ADH* antidiuretic hormone, *CSF* cerebrospinal fluid, *FSH* follicle-stimulating hormone, *GH* growth hormone, *hCG* human chorionic gonadotropin, *LH* luteinizing hormone, *NF1* neurofibromatosis type 1, *TSH* thyroid-stimulating hormone

## Acquired intracranial processes related to glucose metabolism abnormalities: Diabetic ketoacidosis and neonatal hypoglycemia

Type 1 diabetes mellitus (T1DM) results from an immune-mediated destruction of insulin-producing pancreatic β-cells, leading to insulin deficiency. The estimated prevalence of T1DM among children is 3.5 per 1,000 [74]. Individuals with T1DM require life-long exogenous insulin replacement. In the setting of a relative insulin deficiency, patients can develop severe hyperglycemia and diabetic ketoacidosis (DKA), a potentially life-threatening complication. Pediatric patients with known T1DM can develop DKA without hyperglycemia – termed euglycemic DKA – which often occurs in the setting of a vomiting illness or poor oral intake, leading to depletion of glycogen stores and normal or low blood glucose levels, thereby limiting insulin administration. The diagnosis is confirmed by laboratory studies showing a triad of hyperglycemia (>11 mmol/L) or a prior history of diabetes, serum ketosis (beta-hydroxybutyrate ≥3 mmol/L) and/or ketonuria (moderate or large), and acidosis (pH <7.3 or measured bicarbonate <18 mmol/L) with an anion gap >12 [75]. Treatment of DKA includes progressive volume expansion, slow correction of ketoacidosis with an intravenous insulin infusion, and electrolyte repletion [76].

A potentially devastating consequence of DKA is the development of cerebral edema, which typically occurs following treatment initiation. Although cerebral edema occurs in less than 1% of DKA events, it accounts for between 50–90% of pediatric diabetes-related deaths [77]. Warning signs and symptoms of clinically apparent cerebral edema after initiating DKA treatment may include the onset of headache, change in mental status, focal neurological signs including cranial nerve palsy and/or abnormal pupillary responses, emesis, bradycardia, rising blood pressure, or irregular respirations with or without hypoxia [77]. The pathogenesis of cerebral edema in DKA is thought to be a complex interplay of multiple factors including production of vasoactive substances and inflammatory mediators, rapid metabolic and fluid shifts, and breakdown of the blood-brain barrier, all contributing to ischemic-reperfusion injury [77].

**Fig. 23:**
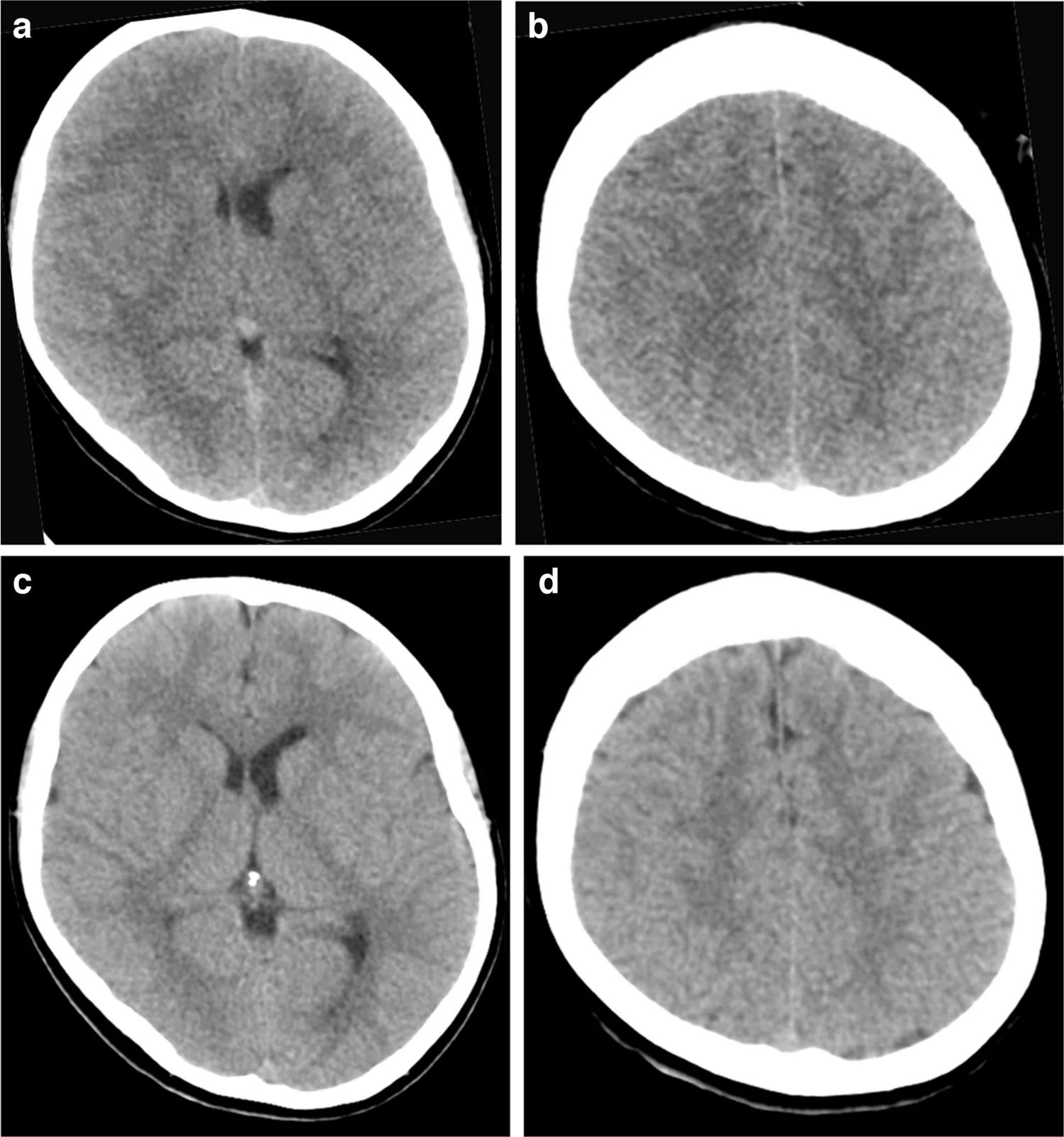
Fourteen-year-old female with diabetic ketoacidosis and cerebral edema. **a, b** Non-contrast head computed tomography images at the level of the (**a**) lateral ventricles and (**b**) vertex show partially effaced slit-like right lateral ventricle and widespread effacement of the cerebral sulci. **c, d** Following treatment with mannitol and hypertonic saline, repeat non-contrast head computed tomography images at the level of the (**c**) lateral ventricles and (**d**) vertex show normalization of the cerebrospinal fluid spaces, confirming decreased or resolved cerebral edema

Concern for cerebral edema may be confirmed by brain imaging with either non-contrast head CT or brain MRI. Findings appreciable on cross-sectional head imaging show narrowing of the ventricles and subarachnoid spaces (Fig. 23). Intracellular cytotoxic edema (i.e., ischemic injury) has not been reported; rather, ADC elevation is exclusively observed in cases of DKA-related cerebral edema [78, 79]. Interestingly, while cerebral edema may only be clinically apparent in 0.3% to 1% of DKA events, ventricular narrowing as evidence of cerebral edema has been observed in children with subclinical DKA [80, 81]. These studies have shown that some degree of cerebral edema is present in more than 50% of children with subclinical DKA based on imaging.

Another topic of abnormal glucose metabolism pertaining to pediatric neuroradiology is congenital hypoglycemia, defined by a sustained, abnormally low blood glucose level in the newborn infant. In healthy term infants, blood glucose values may transiently be as low as 30 mg/dL (1.67 mmol/L) in the first 1 h to 2 h after birth, rising to values similar to older children and adults within 48 h to 72 h with established feeding patterns [82]. This is referred to as transitional hypoglycemia and is common in the healthy newborn. Uncommonly, a more prolonged and severe hypoglycemia may occur. Causes for pathologic hypoglycemia in the newborn include perinatal stress hyperinsulinism, infant of a diabetic mother, inadequate glycogen stores (as seen with prematurity, intrauterine growth restriction, and sepsis), genetic syndromes (for instance, Beckwith-Wiedemann syndrome), congenital hyperinsulinism, pituitary hormone deficiencies (for example, growth hormone and cortisol deficiencies), and inborn errors of metabolism (fatty oxidation defects, gluconeogenic defects, and glycogen storage diseases) [82–85]. Severe and prolonged hypoglycemia in the neonatal population may be associated with seizure activity and abnormal neurologic outcomes, including cortical blindness, intractable epilepsy, developmental delay, hyperactivity and attention difficulties, autism, and microcephaly [86–88].

**Fig. 24:**
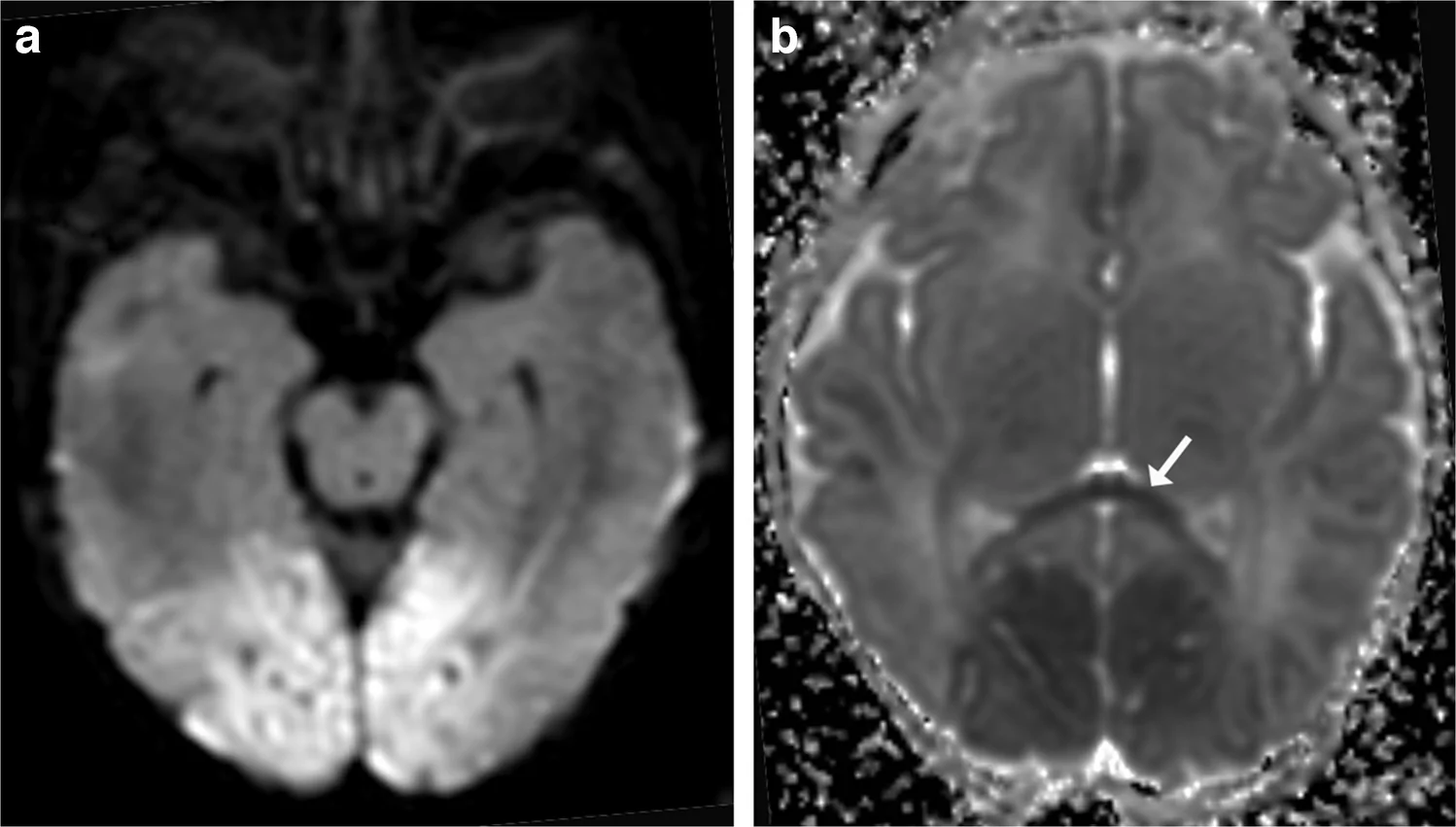
Seven-day-old male infant with hypoglycemia (glucose was less than 10 mg/dL). **a **Axial diffusion-weighted imaging and (**b**) apparent diffusion coefficient map show marked symmetrical cytotoxic edema in the bilateral occipital lobes and in the corpus callosum splenium (*arrow*)

**Fig. 25:**
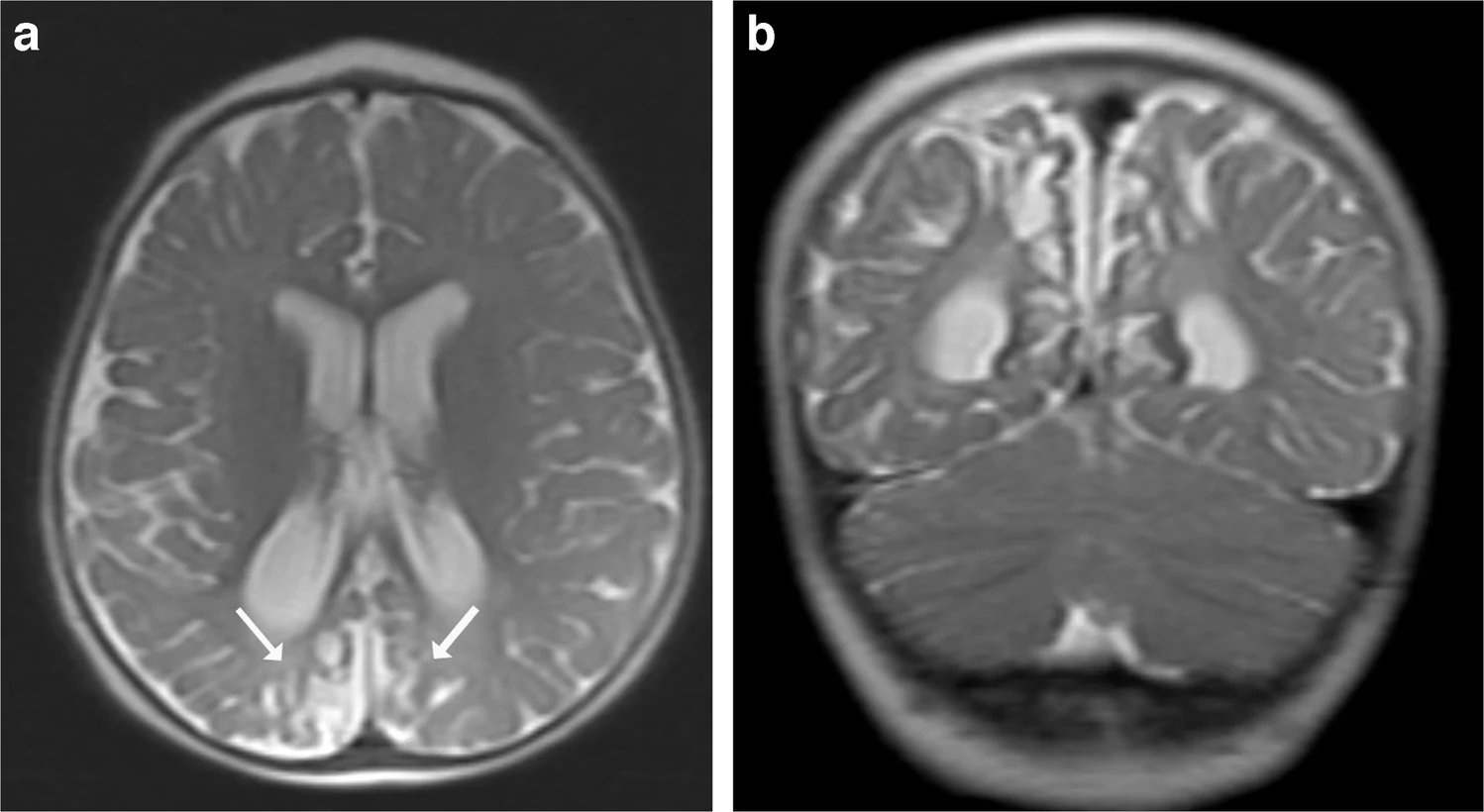
Eleven-month-old male infant with a history of neonatal hypoglycemia. Single-shot fast-spin echo T2-weighted (**a**) axial and (**b**) coronal limited brain magnetic resonance imaging have evidence of occipital cortical or subcortical volume loss, characterized by volume loss and gliosis (*arrows* in **a**)

Diagnostic brain MRI is appropriate for the neonate with hypoglycemia-associated seizures or other neurologic clinical findings and may show abnormalities in both acute and chronic settings. Early in the disease process, typical findings include vasogenic edema (T2-hyperintense) and cytotoxic edema (intense diffusion-weighted signal and low signal on the ADC map) within the posterior cerebral cortices and white matter (most commonly the occipital lobes, but also sometimes involving the parieto-temporal lobes), the corpus callosum, and in the optic radiations (Fig. 24). Deep nuclear gray matter tends to be spared, except in cases of neonatal encephalopathy with mixed etiologies. Specifically, the brain MRI findings and neurologic insults may overlap with hypoxic-ischemic encephalopathy (HIE). Neonates with encephalopathy caused by HIE are at increased risk of depleting their energy stores and developing concurrent hypoglycemia [89]. In cases of neonatal HIE with concomitant hypoglycemia, brain findings include edema in the posterior cerebral white matter, pulvinar, and anterior medial thalamic nuclei [90]. Findings may be unilateral but are usually bilateral. If follow-up imaging is pursued, generalized occipital lobe atrophy may be present (Fig. 25) and may be associated with cortical blindness [87, 91].

Table 3 summarizes these intracranial abnormalities that may result from glucose metabolism disorders.

**Table 3:** Pediatric intracranial findings seen as a consequence of glucose metabolic derangements and their key imaging features

Intracranial pathology	Key features on brain imaging	Endocrine abnormalities & key clinical features
Glucose derangements impacting brain		
Cerebral edema with diabetic ketoacidosis	• Effacement of CSF spaces: narrowing of ventricles and cerebral sulci • No evidence of parenchymal injury on diffusion-weighted imaging; permissive diffusion in cerebral white matter is typically observed	• Diabetic ketoacidosis in type 1 DM patients manifests with hyperglycemia (>11 mmol/L), serum ketosis (beta-hydroxybutyrate ≥3 mmol/L) and/or ketonuria (moderate or large), and acidosis (pH <7.3 or measured bicarbonate <18 mmol/L) with an anion gap >12 • Clinical signs of edema include acute onset of headache, change in mental status, cranial nerve palsy, abnormal pupillary responses, emesis, bradycardia, rising blood pressure, or irregular respirations
Congenital hypoglycemia	• T2-hyperintense signal and restricted diffusion within the posterior cerebral cortices and white matter, corpus callosum, and in optic radiations • Sparing of deep nuclear gray matter when occurring in isolation (may be seen in combined setting of hypoglycemia and hypoxic-ischemic encephalopathy)	• Sustained abnormally low blood glucose levels longer than 72 h in the newborn • Other distinguishing clinical features that may be present depending on underlying etiology: • Fetal growth restriction • Elevated insulin levels and hyperphagia in congenital hyperinsulinism • Overgrowth and hemi-hyperplasia in Beckwith-Wiedemann

*CSF* cerebrospinal fluid, *DM* diabetes mellitus

## Conclusion

Diagnostic imaging with brain MRI is frequently indicated for central endocrinopathies in children. The use of gadolinium as a contrast agent may be required to identify microadenomas and inflammatory conditions, and characterize larger mass lesions including neoplasms occurring in the sella and suprasellar space. Head CT plays a role in the rapid evaluation of some patients with diabetic ketoacidosis and suspected cerebral edema. Management strategies of these disorders by the pediatric endocrinologist are useful for the radiologist to understand for improving communications with referring providers and thereby positively impacting patient management.

## Data Availability

No datasets were generated or analysed during the current study.
